# Association of Country Income Level With the Characteristics and Outcomes of Critically Ill Patients Hospitalized With Acute Kidney Injury and COVID-19

**DOI:** 10.1016/j.ekir.2023.05.015

**Published:** 2023-05-27

**Authors:** Marina Wainstein, Nicholas Spyrison, Danyang Dai, Moji Ghadimi, Jonathan S. Chávez-Iñiguez, Lilia Rizo-Topete, Barbara Wanjiru Citarella, Laura Merson, Jason D. Pole, Rolando Claure-Del Granado, David W. Johnson, Sally Shrapnel, ISARIC Characterization Group, heryl Ann Abdukahil, Nurul Najmee Abdulkadir, Ryuzo Abe, Laurent Abel, Amal Abrous, Lara Absil, Andrew Acker, Shingo Adachi, Diana Adrião, Saleh Al Ageel, Shakeel Ahmed, Kate Ainscough, Eka Airlangga, Tharwat Aisa, Ali Ait Hssain, Younes Ait Tamlihat, Takako Akimoto, Ernita Akmal, Chika Akwani, Eman Al Qasim, Razi Alalqam, Angela Alberti, Tala Al-dabbous, Senthilkumar Alegesan, Marta Alessi, Beatrice Alex, Kévin Alexandre, Abdulrahman Al-Fares, Huda Alfoudri, Imran Ali, Kazali Enagnon Alidjnou, Jeffrey Aliudin, Qabas Alkhafajee, Clotilde Allavena, Nathalie Allou, João Alves, Rita Alves, João Melo Alves, Joana Alves Cabrita, Maria Amaral, Nur Amira, Phoebe Ampaw, Roberto Andini, Claire Andréjak, Andrea Angheben, François Angoulvant, Séverine Ansart, Sivanesen Anthonidass, Massimo Antonelli, Carlos Alexandre Antunes de Brito, Ardiyan Apriyana, Yaseen Arabi, Irene Aragao, Francisco Arancibia, Carolline Araujo, Antonio Arcadipane, Patrick Archambault, Lukas Arenz, Jean-Benoît Arlet, Christel Arnold-Day, Lovkesh Arora, Rakesh Arora, Elise Artaud-Macari, Diptesh Aryal, Angel Asensio, Muhammad Ashraf, Jean Baptiste Assie, Amirul Asyraf, Minahel Atif, Anika Atique, Johann Auchabie, Hugues Aumaitre, Adrien Auvet, Laurène Azemar, Cecile Azoulay, Benjamin Bach, Delphine Bachelet, Claudine Badr, J. Kenneth Baillie, J Kevin Baird, Erica Bak, Agamemnon Bakakos, Nazreen Abu Bakar, Andriy Bal, Mohanaprasanth Balakrishnan, Valeria Balan, Firouzé Bani-Sadr, Renata Barbalho, Nicholas Yuri Barbosa, Wendy S. Barclay, Saef Umar Barnett, Michaela Barnikel, Audrey Barrelet, Cleide Barrigoto, Marie Bartoli, Joaquín Baruch, Romain Basmaci, Muhammad Fadhli Hassin Basri, Denise Battaglini, Jules Bauer, Diego Fernando Bautista Rincon, Abigail Beane, Alexandra Bedossa, Ker Hong Bee, Husna Begum, Sylvie Behilill, Albertus Beishuizen, Aleksandr Beljantsev, David Bellemare, Anna Beltrame, Beatriz Amorim Beltrão, Marine Beluze, Nicolas Benech, Lionel Eric Benjiman, Dehbia Benkerrou, Suzanne Bennett, Binny Benny, Luís Bento, Jan-Erik Berdal, Delphine Bergeaud, Hazel Bergin, José Luis Bernal Sobrino, Giulia Bertoli, Lorenzo Bertolino, Simon Bessis, Sybille Bevilcaqua, Karine Bezulier, Amar Bhatt, Krishna Bhavsar, Claudia Bianco, Farah Nadiah Bidin, Moirangthem Bikram Singh, Felwa Bin Humaid, Mohd Nazlin Bin Kamarudin, Zeno Bisoffi, François Bissuel, Patrick Biston, Laurent Bitker, Jonathan Bitton, Pablo Blanco-Schweizer, Catherine Blier, Frank Bloos, Mathieu Blot, Lucille Blumberg, Filomena Boccia, Laetitia Bodenes, Debby Bogaert, Anne-Hélène Boivin, Isabela Bolaños, Pierre-Adrien Bolze, François Bompart, Diogo Borges, Raphaël Borie, Elisabeth Botelho-Nevers, Lila Bouadma, Olivier Bouchaud, Sabelline Bouchez, Dounia Bouhmani, Damien Bouhour, Kévin Bouiller, Laurence Bouillet, Camile Bouisse, Anne-Sophie Boureau, John Bourke, Maude Bouscambert, Aurore Bousquet, Jason Bouziotis, Bianca Boxma, Marielle Boyer-Besseyre, Maria Boylan, Fernando Augusto Bozza, Axelle Braconnier, Cynthia Braga, Filipa Brás Monteiro, Luca Brazzi, Patrick Breen, Dorothy Breen, David Brewster, Kathy Brickell, Shaunagh Browne, Marjolein Brusse-Keizer, Petra Bryda, Nina Buchtele, Polina Bugaeva, Marielle Buisson, Erlina Burhan, Aidan Burrell, Ingrid G. Bustos, Denis Butnaru, André Cabie, Susana Cabral, Eder Caceres, Cyril Cadoz, Rui Caetano Garcês, Kate Calligy, Jose Andres Calvache, João Camões, Valentine Campana, Paul Campbell, Josie Campisi, Mireia Cantero, Pauline Caraux-Paz, Chiara Simona Cardellino, Sofia Cardoso, Filipe Cardoso, Filipa Cardoso, Nelson Cardoso, Simone Carelli, Nicolas Carlier, Thierry Carmoi, Gayle Carney, Inês Carqueja, Marie-Christine Carret, François Martin Carrier, Ida Carroll, Gail Carson, Maire-Laure Casanova, Mariana Cascão, Siobhan Casey, José Casimiro, Bailey Cassandra, Nidyanara Castanheira, Guylaine Castor-Alexandre, Ivo Castro, Ana Catarino, François-Xavier Catherine, Paolo Cattaneo, Roberta Cavalin, Alexandros Cavayas, Minerva Cervantes-Gonzalez, Anissa Chair, Catherine Chakveatze, Adrienne Chan, Meera Chand, Christelle Chantalat Auger, Jean-Marc Chapplain, Charlotte Charpentier, Julie Chas, Jonathan Samuel Chávez Iñiguez, Anjellica Chen, Yih-Sharng Chen, Léo Chenard, Matthew Pellan Cheng, Antoine Cheret, Thibault Chiarabini, Julian Chica, Suresh Kumar Chidambaram, Leong Chin Tho, Catherine Chirouze, Davide Chiumello, Sung-Min Cho, Bernard Cholley, Marie-Charlotte Chopin, Ting Soo Chow, Yock Ping Chow, Hiu Jian Chua, Jonathan Chua, Jose Pedro Cidade, Barbara Wanjiru Citarella, Anna Ciullo, Jennifer Clarke, Rolando Claure-Del Granado, Sara Clohisey, Perren J. Cobb, Cassidy Codan, Caitriona Cody, Alexandra Coelho, Megan Coles, Jennifer Coles, Gwenhaël Colin, Michael Collins, Sebastiano Maria Colombo, Pamela Combs, Jennifer Connolly, Marie Connor, Anne Conrad, Elaine Conway, Graham S. Cooke, Mary Copland, Hugues Cordel, Amanda Corley, Sabine Cornelis, Alexander Daniel Cornet, Arianne Joy Corpuz, Andrea Cortegiani, Grégory Corvaisier, Camille Couffignal, Sandrine Couffin-Cadiergues, Roxane Courtois, Stéphanie Cousse, Sabine Croonen, Gloria Crowl, Jonathan Crump, Claudina Cruz, Juan Luis Cruz Bermúdez, Jaime Cruz Rojo, Marc Csete, Ailbhe Cullen, Gerard Curley, Elodie Curlier, Colleen Curran, Paula Custodio, Ana da Silva Filipe, Charlene Da Silveira, Al-Awwab Dabaliz, Danyang Dai, Andrew Dagens, Darren Dahly, Heidi Dalton, Jo Dalton, Seamus Daly, Juliana Damas, Nick Daneman, Corinne Daniel, Emmanuelle A. Dankwa, Jorge Dantas, Gillian de Loughry, Etienne De Montmollin, Rafael Freitas de Oliveira França, Rosanna De Rosa, Thushan de Silva, Peter de Vries, David Dean, Alexa Debard, Bianca DeBenedictis, Marie-Pierre Debray, Nathalie DeCastro, William Dechert, Lauren Deconninck, Romain Decours, Eve Defous, Isabelle Delacroix, Eric Delaveuve, Karen Delavigne, Andrea Dell'Amore, Christelle Delmas, Pierre Delobel, Corine Delsing, Elisa Demonchy, Emmanuelle Denis, Dominique Deplanque, Pieter Depuydt, Mehul Desai, Diane Descamps, Mathilde Desvallées, Santi Dewayanti, Pathik Dhanger, Alpha Diallo, Sylvain Diamantis, Fernanda Dias Da Silva, Juan Jose Diaz, Rodrigo Diaz, Kévin Didier, Jean-Luc Diehl, Wim Dieperink, Jérôme Dimet, Vincent Dinot, Fara Diop, Alphonsine Diouf, Yael Dishon, Félix Djossou, Annemarie B. Docherty, Helen Doherty, Maria Donnelly, Christl A. Donnelly, Sean Donohue, Yoann Donohue, Chloe Donohue, Peter Doran, Céline Dorival, Eric D'Ortenzio, James Joshua Douglas, Nathalie Dournon, Triona Downer, Joanne Downey, Mark Downing, Tom Drake, Aoife Driscoll, Murray Dryden, Claudio Duarte Fonseca, Vincent Dubee, François Dubos, Alexandre Ducancelle, Toni Duculan, Susanne Dudman, Abhijit Duggal, Paul Dunand, Jake Dunning, Mathilde Duplaix, Emanuele Durante-Mangoni, Lucian Durham, Bertrand Dussol, Juliette Duthoit, Xavier Duval, Anne Margarita Dyrhol-Riise, Sim Choon Ean, Marco Echeverria-Villalobos, Eleanor Edwards, Siobhan Egan, Mohammed El Sanharawi, Subbarao Elapavaluru, Brigitte Elharrar, Philippine Eloy, Tarek Elshazly, Isabelle Enderle, Tomoyuki Endo, Chan Chee Eng, Ilka Engelmann, Vincent Enouf, Olivier Epaulard, Martina Escher, Mariano Esperatti, Hélène Esperou, Catarina Espírito Santo, Marina Esposito-Farese, João Estevão, Manuel Etienne, Nadia Ettalhaoui, Anna Greti Everding, Mirjam Evers, Marc Fabre, Isabelle Fabre, Amna Faheem, Arabella Fahy, Cameron J. Fairfield, Pedro Faria, Hanan Fateena, Arie Zainul Fatoni, Karine Faure, Raphaël Favory, Mohamed Fayed, Niamh Feely, Laura Feeney, Jorge Fernandes, Marília Andreia Fernandes, Susana Fernandes, François-Xavier Ferrand, Eglantine Ferrand Devouge, Joana Ferrão, Mário Ferraz, Sílvia Ferreira, Bernardo Ferreira, Benigno Ferreira, Nicolas Ferriere, Céline Ficko, Claudia Figueiredo-Mello, Juan Fiorda, Thomas Flament, Emily Flanagan, Clara Flateau, Tom Fletcher, Aline-Marie Florence, Letizia Lucia Florio, Deirdre Flynn, Jean Foley, Victor Fomin, Tatiana Fonseca, Simon Forsyth, Denise Foster, Giuseppe Foti, Erwan Fourn, Robert A. Fowler, Marianne Fraher, Diego Franch-Llasat, John F. Fraser, Christophe Fraser, Marcela Vieira Freire, Ana Freitas Ribeiro, Caren Friedrich, Ricardo Fritz, Stéphanie Fry, Nora Fuentes, Masahiro Fukuda, Argin G, Valérie Gaborieau, Rostane Gaci, Massimo Gagliardi, Jean-Charles Gagnard, Amandine Gagneux-Brunon, Sérgio Gaião, Linda Gail Skeie, Phil Gallagher, Carrol Gamble, Yasmin Gani, Arthur Garan, Rebekha Garcia, Noelia García Barrio, Esteban Garcia-Gallo, Denis Garot, Valérie Garrait, Nathalie Gault, Aisling Gavin, Anatoliy Gavrylov, Alexandre Gaymard, Johannes Gebauer, Eva Geraud, Louis Gerbaud Morlaes, Nuno Germano, Jade Ghosn, Marco Giani, Jess Gibson, Tristan Gigante, Morgane Gilg, Elaine Gilroy, Guillermo Giordano, Michelle Girvan, Valérie Gissot, Daniel Glikman, Petr Glybochko, Eric Gnall, François Goehringer, Jean-Christophe Goffard, Jin Yi Goh, Jonathan Golob, Marie Gominet, Alicia Gonzalez, Patricia Gordon, Isabelle Gorenne, Conor Gormley, Laure Goubert, Cécile Goujard, Tiphaine Goulenok, Margarite Grable, Edward Wilson Grandin, Pascal Granier, Giacomo Grasselli, Christopher A. Green, William Greenhalf, Segolène Greffe, Domenico Luca Grieco, Matthew Griffee, Fiona Griffiths, Ioana Grigoras, Albert Groenendijk, Heidi Gruner, Yusing Gu, Jérémie Guedj, Martin Guego, Dewi Guellec, Daniela Guerreiro, Romain Guery, Anne Guillaumot, Laurent Guilleminault, Maisa Guimarães de Castro, Thomas Guimard, Marieke Haalboom, Daniel Haber, Hannah Habraken, Ali Hachemi, Nadir Hadri, Sheeba Hakak, Adam Hall, Matthew Hall, Sophie Halpin, Ansley Hamer, Raph L. Hamers, Rebecca Hamidfar, Terese Hammond, Naomi Hammond, Lim Yuen Han, Kok Wei Hao, Hayley Hardwick, Ewen M. Harrison, Janet Harrison, Alan Hartman, Junaid Hashmi, Ailbhe Hayes, Leanne Hays, Jan Heerman, Lars Heggelund, Ross Hendry, Martina Hennessy, Maxime Hentzien, Diana Hernandez, Andrew Hershey, Liv Hesstvedt, Eibhlin Higgins, Dawn Higgins, Rupert Higgins, Rita Hinchion, Samuel Hinton, Hiroaki Hiraiwa, Hikombo Hitoto, Antonia Ho, Yi Bin Ho, Alexandre Hoctin, Isabelle Hoffmann, Wei Han Hoh, Oscar Hoiting, Rebecca Holt, Jan Cato Holter, Peter Horby, Kota Hoshino, Ikram Houas, Catherine L. Hough, Stuart Houltham, Jimmy Ming-Yang Hsu, Jean-Sébastien Hulot, Abby Hurd, Samreen Ijaz, M. Arfan Ikram, Hajnal-Gabriela Illes, Patrick Imbert, Hugo Inácio, Yun Sii Ing, Mariachiara Ippolito, Sarah Isgett, Tiago Isidoro, Nadiah Ismail, Margaux Isnard, Junji Itai, Daniel Ivulich, Danielle Jaafar, Salma Jaafoura, Julien Jabot, Clare Jackson, Pierre Jaquet, Waasila Jassat, Coline Jaud-Fischer, Stéphane Jaureguiberry, Florence Jego, Anilawati Mat Jelani, Synne Jenum, Ong Yiaw Joe, Ruth N. Jorge García, Mark Joseph, Cédric Joseph, Mercé Jourdain, Philippe Jouvet, Anna Jung, Dafsah Juzar, Ouifiya Kafif, Florentia Kaguelidou, Neerusha Kaisbain, Thavamany Kaleesvran, Sabina Kali, Smaragdi Kalomoiri, Muhammad Aisar Ayadi Kamaluddin, Zul Amali Che Kamaruddin, Nadiah Kamarudin, Darshana Hewa Kandamby, Chris Kandel, Kong Yeow Kang, Pratap Karpayah, Christiana Kartsonaki, Daisuke Kasugai, Anant Kataria, Kevin Katz, Aasmine Kaur, Christy Kay, Lamees Kayyali, Seán Keating, Claire Kelly, Yvelynne Kelly, Andrea Kelly, Niamh Kelly, Aoife Kelly, Sadie Kelly, Maeve Kelsey, Ryan Kennedy, Kalynn Kennon, Maeve Kernan, Younes Kerroumi, Sharma Keshav, Imrana Khalid, Antoine Khalil, Coralie Khan, Irfan Khan, Michelle E. Kho, Saye Khoo, Ryan Khoo, Denisa Khoo, Khor How Kiat, Yuri Kida, Peter Kiiza, Beathe Kiland Granerud, Anders Benjamin Kildal, Jae Burm Kim, Antoine Kimmoun, Nobuya Kitamura, Paul Klenerman, Rob Klont, Gry Kloumann Bekken, Stephen R. Knight, Mamoru Komatsu, Volkan Korten, Arsène Kpangon, Karolina Krawczyk, Sudhir Krishnan, Vinothini Krishnan, Oksana Kruglova, Deepali Kumar, Ganesh Kumar, Bharath Kumar Tirupakuzhi Vijayaraghavan, Pavan Kumar Vecham, Dinesh Kuriakose, Ethan Kurtzman, Demetrios Kutsogiannis, Galyna Kutsyna, Konstantinos Kyriakoulis, Marie Lachatre, Marie Lacoste, John G. Laffey, Nadhem Lafhej, Marie Lagrange, Fabrice Laine, Olivier Lairez, Antonio Lalueza, Marc Lambert, Marie Langelot-Richard, Vincent Langlois, Eka Yudha Lantang, Marina Lanza, Cédric Laouénan, Samira Laribi, Delphine Lariviere, Stéphane Lasry, Odile Launay, Didier Laureillard, Yoan Lavie-Badie, Andy Law, Teresa Lawrence, Cassie Lawrence, Minh Le, Clément Le Bihan, Cyril Le Bris, Georges Le Falher, Lucie Le Fevre, Quentin Le Hingrat, Marion Le Maréchal, Soizic Le Mestre, Gwenaël Le Moal, Vincent Le Moing, Hervé Le Nagard, Paul Le Turnier, Todd C. Lee, Su Hwan Lee, James Lee, Jennifer Lee, Heng Gee Lee, Biing Horng Lee, Yi Lin Lee, Gary Leeming, Laurent Lefebvre, Bénédicte Lefebvre, Benjamin Lefèvre, Sylvie LeGac, Jean-Daniel Lelievre, François Lellouche, Adrien Lemaignen, Véronique Lemee, Anthony Lemeur, Gretchen Lemmink, Ha Sha Lene, Michela Leone, Marc Leone, Quentin Lepiller, François-Xavier Lescure, Olivier Lesens, Mathieu Lesouhaitier, Amy Lester-Grant, Sophie Letrou, Yves Levy, Bruno Levy, Claire Levy-Marchal, Erwan L'Her, Gianluigi Li Bassi, Janet Liang, Geoffrey Liegeon, Wei Shen Lim, Kah Chuan Lim, Chantre Lima, Bruno Lina, Lim Lina, Andreas Lind, Guillaume Lingas, Sylvie Lion-Daolio, Keibun Liu, Marine Livrozet, Patricia Lizotte, Navy Lolong, Leong Chee Loon, Diogo Lopes, Dalia Lopez-Colon, Anthony L. Loschner, Paul Loubet, Bouchra Loufti, Guillame Louis, Silvia Lourenco, Lee Low, Marije Lowik, Jia Shyi Loy, Jean Christophe Lucet, Carlos Lumbreras Bermejo, Carlos M. Luna, Olguta Lungu, Miles Lunn, Liem Luong, Nestor Luque, Dominique Luton, Ruth Lyons, Olavi Maasikas, Oryane Mabiala, Sara Machado, Moïse Machado, Gabriel Macheda, Hashmi Madiha, Guillermo Maestro de la Calle, Rafael Mahieu, Sophie Mahy, Lars S. Maier, Mylène Maillet, Thomas Maitre, Maximilian Malfertheiner, Nadia Malik, Paddy Mallon, Denis Malvy, Victoria Manda, Laurent Mandelbrot, Frank Manetta, Julie Mankikian, Edmund Manning, Aldric Manuel, Ceila Maria Sant`Ana Malaque, Flávio Marino, Samuel Markowicz, Ana Marques, Brian Marsh, Laura Marsh, Megan Marshal, John Marshall, Celina Turchi Martelli, Emily Martin, Guillaume Martin-Blondel, F. Eduardo Martinez, Ignacio Martin-Loeches, Martin Martinot, João Martins, Ana Martins, Caroline Martins Rego, Gennaro Martucci, Olga Martynenko, Eva Miranda Marwali, Marsilla Marzukie, David Maslove, Sabina Mason, Mohd Basri Mat Nor, Moshe Matan, Daniel Mathieu, Mathieu Mattei, Laurence Maulin, Michael Maxwell, Thierry Mazzoni, Lisa Mc Sweeney, Colin McArthur, Peter McCanny, Anne McCarthy, Aine McCarthy, Colin McCloskey, Rachael McConnochie, Sherry McDermott, Sarah E. McDonald, Aine McElroy, Samuel McElwee, Victoria McEneany, Natalie McEvoy, Allison McGeer, Chris McKay, Johnny McKeown, Kenneth A. McLean, Bairbre McNicholas, Elaine McPartlan, Edel Meaney, Cécile Mear-Passard, Maggie Mechlin, Omar Mehkri, Ferruccio Mele, Kusum Menon, France Mentré, Alexander J. Mentzer, Noémie Mercier, Emmanuelle Mercier, Antoine Merckx, Mayka Mergeay-Fabre, Blake Mergler, Laura Merson, António Mesquita, Osama Metwally, Agnès Meybeck, Dan Meyer, Alison M. Meynert, Vanina Meysonnier, Amina Meziane, Mehdi Mezidi, Céline Michelanglei, Isabelle Michelet, Efstathia Mihelis, Vladislav Mihnovit, Jennene Miller, Nor Arisah Misnan, Tahira Jamal Mohamed, Nik Nur Eliza Mohamed, Asma Moin, Elena Molinos, Brenda Molloy, Sinead Monahan, Mary Mone, Agostinho Monteiro, Claudia Montes, Giorgia Montrucchio, Shona C. Moore, Sarah Moore, Lina Morales Cely, Lucia Moro, Catherine Motherway, Ana Motos, Hugo Mouquet, Clara Mouton Perrot, Julien Moyet, Caroline Mudara, Ng Yong Muh, Dzawani Muhamad, Jimmy Mullaert, Fredrik Müller, Karl Erik Müller, Daniel Munblit, Aisling Murphy, Lorna Murphy, Patrick Murray, Marlène Murris, Srinivas Murthy, Himed Musaab, Gugapriyaa Muyandy, Dimitra Melia Myrodia, Dave Nagpal, Alex Nagrebetsky, Mangala Narasimhan, Nageswaran Narayanan, Nadège Neant, Coca Necsoi, Nikita Nekliudov, Raul Neto, Emily Neumann, Pauline Yeung Ng, Wing Yiu Ng, Anthony Nghi, Duc Nguyen, Orna Ni Choileain, Niamh Ni Leathlobhair, Alistair D. Nichol, Prompak Nitayavardhana, Stephanie Nonas, Nurul Amani Mohd Noordin, Marion Noret, Nurul Faten Izzati Norharizam, Lisa Norman, Alessandra Notari, Mahdad Noursadeghi, Saad Nseir, Dwi Utomo Nusantara, Elsa Nyamankolly, Fionnuala O. Brien, Annmarie O. Callaghan, Annmarie O'Callaghan, Giovanna Occhipinti, Derbrenn OConnor, Takayuki Ogura, Sophie O'Halloran, Katie O'Hearn, Shinichiro Ohshimo, João Oliveira, Piero L. Olliaro, Conar O'Neil, David S.Y. Ong, Jee Yan Ong, Wilna Oosthuyzen, Anne Opavsky, Peter Openshaw, Claudia Milena Orozco-Chamorro, Jamel Ortoleva, Javier Osatnik, Linda O'Shea, Miriam O'Sullivan, Siti Zubaidah Othman, Nadia Ouamara, Rachida Ouissa, Eric Oziol, Maïder Pagadoy, Justine Pages, Massimo Palmarini, Giovanna Panarello, Prasan Kumar Panda, Lai Hui Pang, Mauro Panigada, Nathalie Pansu, Aurélie Papadopoulos, Rachael Parke, Jérémie Pasquier, Bruno Pastene, Fabian Patauner, Mohan Dass Pathmanathan, Patricia Patricio, Juliette Patrier, Lisa Patterson, Mical Paul, Christelle Paul, Jorge Paulos, William A. Paxton, Jean-François Payen, Sandra L. Peake, Kalaiarasu Peariasamy, Miguel Pedrera Jiménez, Giles J. Peek, Florent Peelman, Nathan Peiffer-Smadja, Vincent Peigne, Mare Pejkovska, Paolo Pelosi, Ithan D. Peltan, Rui Pereira, Daniel Perez, Luis Periel, Thomas Perpoint, Antonio Pesenti, Vincent Pestre, Lenka Petrou, Michele Petrovic, Ventzislava Petrov-Sanchez, Frank Olav Pettersen, Gilles Peytavin, Scott Pharand, Michael Piagnerelli, Walter Picard, Olivier Picone, Carola Pierobon, Djura Piersma, Carlos Pimentel, Valentine Piquard, Catarina Pires, Isabelle Pironneau, Lionel Piroth, Ayodhia Pitaloka, Chiara Piubelli, Riinu Pius, Laurent Plantier, Hon Shen Png, Julien Poissy, Ryadh Pokeerbux, Sergio Poli, Georgios Pollakis, Diane Ponscarme, Diego Bastos Porto, Andra-Maris Post, Douwe F. Postma, Pedro Povoa, Diana Póvoas, Jeff Powis, Sofia Prapa, Sébastien Preau, Christian Prebensen, Jean-Charles Preiser, Anton Prinssen, Lucia Proença, Sravya Pudota, Oriane Puéchal, Bambang Pujo Semedi, Gregory Purcell, Luisa Quesada, Víctor Quirós González, Else Quist-Paulsen, Mohammed Quraishi, Fadi Qutishat, Christian Rabaud, Aldo Rafael, Marie Rafiq, Mutia Rahardjani, Amir Kamel Rahimi, Rozanah Abd Rahman, Ahmad Kashfi Haji Ab Rahman, Fernando Rainieri, Giri Shan Rajahram, Nagarajan Ramakrishnan, José Ramalho, Ahmad Afiq Ramli, Blandine Rammaert, Grazielle Viana Ramos, Ritika Ranjan, Christophe Rapp, Menaldi Rasmin, Indrek Rätsep, Tharmini Ravi, Andre Real, Stanislas Rebaudet, Sarah Redl, Brenda Reeve, Liadain Reid, Dag Henrik Reikvam, Renato Reis, Martine Remy, Hongru Ren, Anne-Sophie Resseguier, Matthieu Revest, Oleksa Rewa, Tiago Reyes, Luis Felipe Reyes, Maria Ines Ribeiro, David Richardson, Denise Richardson, Laurent Richier, Siti Nurul Atikah Ahmad Ridzuan, Ana L. Rios, Asgar Rishu, Patrick Rispal, Karine Risso, Nicholas Rizer, Chiara Robba, André Roberto, Stephanie Roberts, David L. Robertson, Olivier Robineau, Ferran Roche-Campo, Paola Rodari, Simão Rodeia, Bernhard Roessler, Pierre-Marie Roger, Amanda Rojek, Juliette Romaru, Roberto Roncon-Albuquerque, Mélanie Roriz, Manuel Rosa-Calatrava, Michael Rose, Dorothea Rosenberger, Andrea Rossanese, Matteo Rossetti, Bénédicte Rossignol, Patrick Rossignol, Stella Rousset, Carine Roy, Benoît Roze, Clark D. Russell, Maria Ryan, Maeve Ryan, Steffi Ryckaert, Aleksander Rygh Holten, Isabela Saba, Musharaf Sadat, Valla Sahraei, Maximilien Saint-Gilles, Pranya Sakiyalak, Leonardo Salazar, Gabriele Sales, Stéphane Sallaberry, Charlotte Salmon Gandonniere, Hélène Salvator, Emely Sanchez, Olivier Sanchez, Angel Sanchez-Miralles, Vanessa Sancho-Shimizu, Gyan Sandhu, Zulfiqar Sandhu, Pierre-François Sandrine, Oana Sandulescu, Marlene Santos, Shirley Sarfo-Mensah, Iam Claire E. Sarmiento, Benjamine Sarton, Sree Satyapriya, Rumaisah Satyawati, Egle Saviciute, Yen Tsen Saw, Justin Schaffer, Tjard Schermer, Arnaud Scherpereel, Marion Schneider, Stephan Schroll, Michael Schwameis, Janet T. Scott, James Scott-Brown, Nicholas Sedillot, Tamara Seitz, Mageswari Selvarajoo, Caroline Semaille, Malcolm G. Semple, Rasidah Bt Senian, Eric Senneville, Claudia Sepulveda, Filipa Sequeira, Tânia Sequeira, Ary Serpa Neto, Pablo Serrano Balazote, Ellen Shadowitz, Syamin Asyraf Shahidan, Mohammad Shamsah, Anuraj Shankar, Shaikh Sharjeel, Pratima Sharma, Catherine A. Shaw, Victoria Shaw, John Robert Sheenan, Haixia Shi, Nobuaki Shime, Hiroaki Shimizu, Keiki Shimizu, Sally Shrapnel, Hoi Ping Shum, Nassima Si Mohammed, Ng Yong Siang, Jeanne Sibiude, Atif Siddiqui, Louise Sigfrid, Piret Sillaots, Catarina Silva, Rogério Silva, Maria Joao Silva, Benedict Sim Lim Heng, Wai Ching Sin, Punam Singh, Budha Charan Singh, Pompini Agustina Sitompul, Karisha Sivam, Vegard Skogen, Sue Smith, Benjamin Smood, Coilin Smyth, Michelle Smyth, Morgane Snacken, Dominic So, Tze Vee Soh, Joshua Solomon, Tom Solomon, Emily Somers, Agnès Sommet, Rima Song, Myung Jin Song, Tae Song, Jack Song Chia, Albert Sotto, Edouard Soum, Marta Sousa, Ana Chora Sousa, Maria Sousa Uva, Vicente Souza-Dantas, Alexandra Sperry, Elisabetta Spinuzza, Shiranee Sriskandan, Sarah Stabler, Thomas Staudinger, Stephanie-Susanne Stecher, Trude Steinsvik, Ymkje Stienstra, Birgitte Stiksrud, Eva Stolz, Amy Stone, Adrian Streinu-Cercel, Anca Streinu-Cercel, Ami Stuart, David Stuart, Richa Su, Decy Subekti, Gabriel Suen, Jacky Y. Suen, Prasanth Sukumar, Asfia Sultana, Charlotte Summers, Dubravka Supic, Deepashankari Suppiah, Magdalena Surovcová, Suwarti; Andrey Svistunov, Sarah Syahrin, Konstantinos Syrigos, Jaques Sztajnbok, Konstanty Szuldrzynski, Shirin Tabrizi, Fabio S. Taccone, Lysa Tagherset, Shahdattul Mawarni Taib, Sara Taleb, Jelmer Talsma, Maria Lawrensia Tampubolon, Kim Keat Tan, Yan Chyi Tan, Hiroyuki Tanaka, Taku Tanaka, Hayato Taniguchi, Coralie Tardivon, Pierre Tattevin, M Azhari Taufik, Hassan Tawfik, Richard S. Tedder, Tze Yuan Tee, João Teixeira, Marie-Capucine Tellier, Sze Kye Teoh, Vanessa Teotonio, François Téoulé, Pleun Terpstra, Olivier Terrier, Nicolas Terzi, Hubert Tessier-Grenier, Adrian Tey, Alif Adlan Mohd Thabit, Zhang Duan Tham, Suvintheran Thangavelu, Elmi Theron, Vincent Thibault, Simon-Djamel Thiberville, Benoît Thill, Jananee Thirumanickam, Shaun Thompson, David Thomson, Emma C. Thomson, Mathew Thorpe, Surain Raaj Thanga Thurai, Ryan S. Thwaites, Paul Tierney, Vadim Tieroshyn, Peter S. Timashev, Jean-François Timsit, Noémie Tissot, Fiona Toal, Jordan Zhien Yang Toh, Maria Toki, Kristian Tonby, Sia Loong Tonnii, Marta Torre, Antoni Torres, Hernando Torres-Zevallos, Michael Towers, Tony Trapani, Cécile Tromeur, Ioannis Trontzas, Tiffany Trouillon, Jeanne Truong, Christelle Tual, Sarah Tubiana, Helen Tuite, Alexis F. Turgeon, Jean-Marie Turmel, Lance C.W. Turtle, Anders Tveita, Pawel Twardowski, Andrew Udy, Roman Ullrich, Alberto Uribe, Asad Usman, Timothy M. Uyeki, Cristinava Vajdovics, Luís Val-Flores, Amélie Valran, Stijn Van de Velde, Marcel van den Berge, Job van der Palen, Paul van der Valk, Nicky Van Der Vekens, Peter Van der Voort, Sylvie Van Der Werf, Laura van Gulik, Jarne Van Hattem, Carolien van Netten, Ilonka van Veen, Noémie Vanel, Henk Vanoverschelde, Pooja Varghese, Michael Varrone, Shoban Raj Vasudayan, Charline Vauchy, Shaminee Veeran, Aurélie Veislinger, Sebastian Vencken, Sara Ventura, Annelies Verbon, José Ernesto Vidal, César Vieira, Joy Ann Villanueva, Andrea Villoldo, Benoit Visseaux, Chiara Vitiello, Harald Vonkeman, Fanny Vuotto, Noor Hidayu Wahab, Suhaila Abdul Wahab, Nadirah Abdul Wahid, Marina Wainstein, Laura Walsh, Chih-Hsien Wang, Steve Webb, Jia Wei, Tan Pei Wen, Sanne Wesselius, Murray Wham, Bryan Whelan, Nicole White, Paul Henri Wicky, Aurélie Wiedemann, Surya Otto Wijaya, Keith Wille, Sue Willems, Bailey Williams, Virginie Williams, Patricia J. Williams, Evert-Jan Wils, Jessica Wittman, Calvin Wong, Xin Ci Wong, Yew Sing Wong, Teck Fung Wong, Gan Ee Xian, Lim Saio Xian, Pei Xuan Kuan, Ioannis Xynogalas, Siti Rohani Binti Mohd Yakop, Masaki Yamazaki, Elizabeth Yarad, Yazdan Yazdanpanah, Nicholas Yee Liang Hing, Cécile Yelnik, Chian Hui Yeoh, Stephanie Yerkovich, Toshiki Yokoyama, Hodane Yonis, Obada Yousif, Akram Zaaqoq, Marion Zabbe, Masliza Zahid, Maram Zahran, Nor Zaila Binti Zaidan, Maria Zambon, Miguel Zambrano, Alberto Zanella, Nurul Zaynah, Hiba Zayyad, Alexander Zoufaly, David Zucman

**Affiliations:** 1 Faculty of Medicine, University of Queensland, Brisbane, Australia; 2 West Moreton Kidney Health Service, Brisbane, Queensland, Australia; 3 International Severe Acute Respiratory and emerging Infections Consortium (ISARIC), Pandemic Sciences Institute, University of Oxford, Oxford, UK; 4 School of Mathematics and Physics, University of Queensland, Brisbane, Australia; 5 Centre for Health Services Research, Faculty of Medicine, University of Queensland, Brisbane, Australia; 6 Servicio de Nefrología, Hospital Civil de Guadalajara, Guadalajara, México; 7 Autonomous University of Nuevo León, San Nicolas de los Garza, México; 8 Division of Nephrology Hospital Obrero No 2 - CNS, Cochabamba, Bolivia; 9 Universidad Mayor de San Simon, School of Medicine, Cochabamba, Bolivia; 10 Metro South Kidney and Transplant Services (MSKATS), Princess Alexandra Hospital, Brisbane, Queensland, Australia; 11 Centre for Kidney Disease Research, University of Queensland at Princess Alexandra Hospital, Brisbane, Queensland, Australia; 12 Translational Research Institute, Brisbane, Queensland, Australia; 13 ARC Centre of Excellence for Engineered Quantum Systems, School of Mathematics and Physics, University of Queensland, Queensland, Australia

**Keywords:** acute kidney injury, community-acquired AKI, country income, COVID-19, dialysis, in-hospital death

## Abstract

**Introduction:**

Acute kidney injury (AKI) has been identified as one of the most common and significant problems in hospitalized patients with COVID-19. However, studies examining the relationship between COVID-19 and AKI in low- and low-middle income countries (LLMIC) are lacking. Given that AKI is known to carry a higher mortality rate in these countries, it is important to understand differences in this population.

**Methods:**

This prospective, observational study examines the AKI incidence and characteristics of 32,210 patients with COVID-19 from 49 countries across all income levels who were admitted to an intensive care unit during their hospital stay.

**Results:**

Among patients with COVID-19 admitted to the intensive care unit, AKI incidence was highest in patients in LLMIC, followed by patients in upper-middle income countries (UMIC) and high-income countries (HIC) (53%, 38%, and 30%, respectively), whereas dialysis rates were lowest among patients with AKI from LLMIC and highest among those from HIC (27% vs. 45%). Patients with AKI in LLMIC had the largest proportion of community-acquired AKI (CA-AKI) and highest rate of in-hospital death (79% vs. 54% in HIC and 66% in UMIC). The association between AKI, being from LLMIC and in-hospital death persisted even after adjusting for disease severity.

**Conclusions:**

AKI is a particularly devastating complication of COVID-19 among patients from poorer nations where the gaps in accessibility and quality of healthcare delivery have a major impact on patient outcomes.

## Graphical abstract

**Figure:**
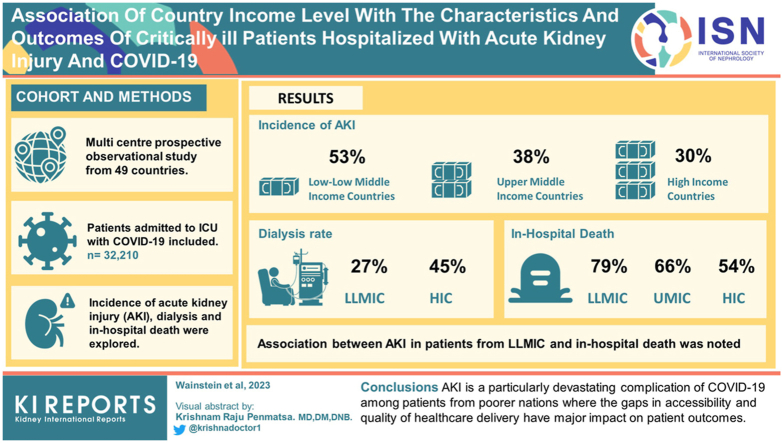

Since its beginnings in December of 2019, SARS-CoV-2 has infected >680 million people and claimed the lives of 6.8 million more.^1^ It has done so indiscriminately of geography, age, race, or socioeconomic status, positioning itself as one of the most consequential and tragic health crisis of this century. Beyond the idiosyncratic and situational factors that have allowed this virus to spread unhindered across entire continents, much of the observed morbidity and mortality has resulted from its capacity for multiorgan involvement followed by rapid deterioration. In this context, AKI has been identified as one of the most common and significant problems in hospitalized patients with COVID-19.^2^, ^3^, ^4^ Whether through direct viral invasion, hemodynamic shifts, or local inflammatory and thrombotic changes,^5^ AKI has been shown to be a catalyst for longer admission times, increased need for ICU level care and in-hospital death.^2^^,^^6^^,^^7^ Thus far, most of what we know about AKI in COVID-19 comes from observational studies of patients from HIC. Although largely unaccounted for in the scientific literature, COVID-19 has had a decimating effect on countries and regions with fragmented healthcare systems and large portions of their population living in poverty.^8^

We know from the ISN 0by25 Global Snapshot of AKI study that, in patients from LLMIC, AKI is predominantly community-acquired and is associated with a higher rate of mortality.^9^^,^^10^ The Global Kidney Health Atlas has shown that access to kidney replacement therapies is critically conditioned by socioeconomic factors and not just clinical indication in resource-limited areas.^11^ In patients with COVID-19, the few studies from low and middle income countries have supported these general observations.^12^^,^^13^ However, their small sample sizes and restricted geographic representation limit the generalizability and robustness of their conclusions.

To further examine this relationship, we undertook the first multinational study comparing the characteristics and outcomes of AKI in patients hospitalized with COVID-19 stratified by country income level. We hypothesized that a larger proportion of CA-AKI and higher AKI-associated mortality would be observed among patients from LLMIC compared with those from higher income countries. Given the resource limitations experienced by lower income countries, we also hypothesized that acute dialysis would be less prevalent among patients with AKI from LLMIC.

## Methods

### Study Design

The International Severe Acute Respiratory and Emerging Infection Consortium - World Health Organization Clinical Characterization Protocol for Severe Emerging Infections provided a framework for prospective, observational data collection on hospitalized patients affected by pathogens of public health interest.^14^ The protocol, case report forms (CRFs) and study information are available online (https://isaric.org/research/covid-19-clinical-research-resources), of which only the core CRF was used in this study.^15^ These CRFs were developed to standardize clinical data collection on patients admitted with suspected or confirmed COVID-19 and have been widely used since the start of the pandemic.^2^^,^^16^ Collection of serum creatinine measurements across all sites was not time-standardized and the frequency of collection was left to the discretion of each site.

This observational study required no change to clinical management and encouraged patient enrolment in other research projects. Protocol and consent forms are available at https://isaric.net/ccp/. Whereas written consent was obtained in most cases, for some sites the local regulators and ethics committees approved oral consent, or waiver of consent, in the context of the pandemic.

The Clinical Characterization Protocol was approved by the World Health Organization Ethics Review Committee (RPC571 and RPC572, 25 April 2013). Ethical approval was obtained for each participating country and site according to local requirements (Supplementary Statement S1). We reported the study in accordance with the STROBE (Strengthening the Reporting of Observational Studies in Epidemiology) reporting guideline (Supplementary Table S1).^17^

### Study Population

#### Inclusion and Exclusion Criteria

All individuals in the International Severe Acute Respiratory and Emerging Infection Consortium - World Health Organization Clinical Characterization Protocol database with clinically diagnosed or laboratory-confirmed SARS-CoV-2 infection and available hospital laboratory data admitted to hospital from January 30, 2020, to September 1, 2022 (criteria for clinical diagnosis in Supplementary Table S2) were included in this analysis. Data were collected and analyzed for the duration of a patient’s admission.

Patients younger than 18 years of age and those on maintenance kidney replacement therapies (dialysis or kidney transplantation) were excluded first followed by patients who had not been admitted to the ICU at any point during their hospital stay. Given that a significant proportion of patient data was recruited through the Critical Care Asia-consortium, >98% of the LLMIC participants experienced an ICU admission during their stay. The exclusion was made to reduce this known bias. Patients with fewer than 2 serum creatinine (sCr) measurements and those with incomplete or unreliable demographic or laboratory data were excluded next. Finally, patients without an admission outcome which included death, discharge, or transfer, either because one was not reported in the CRF or the patient was lost to follow-up, were labeled as “lost to follow-up” and excluded from the analysis.

Patients in the final analysis cohort were then divided according to country income level based on the World Bank classification (https://data.worldbank.org/country): LLMIC, UMIC, and HIC. Patients from LLMIC were placed in a single category because of the small patient numbers in low-income countries.

### AKI Incidence and Time to Peak AKI

AKI was identified biochemically using the sCr criteria of the Kidney Disease Improving Global Outcomes definition of AKI, which requires a patient to have an increase in sCr by ≥ 26.5 μmol/l within 48 hours or an increase to >1.5 times the baseline sCr within 7 days.^18^ Urine volume criteria were not used because urine volume was not routinely collected in the CRF. Time to peak AKI from hospital admission and the respective counts for each day were compared by visual inspection of histograms using the first day that a peak stage was reached. The first occurrence of AKI was classified as CA-AKI if it was detected within the first 48 hours of admission and hospital-acquired AKI if it developed thereafter.

From the prespecified data collected in the CRF, information was obtained on patients’ comorbidities and preadmission medications as well as signs, symptoms, observations, and laboratory results on admission to hospital. Information collected during the admission included acute treatments, complications, and outcomes. Outcomes included discharge, transfer to another hospital or in-hospital death. Definitions of all collected variables are provided in Supplementary Table S3. Only those variables with <20% missingness, in any income country group, were analyzed.

### Statistical Analysis

For continuous variables, characteristics were reported as medians and interquartile ranges (IQR). For categorical variables, counts and percentages were reported. All statistical tests were carried out as pairwise independent samples comparisons. Because of the number of statistical tests conducted, a conservative Bonferroni adjusted significance level of $\alpha_{b}$ 5 × 10^-5^ was used to limit the study wide probability of a type I error.^19^ For continuous variables, the Mann-Whitney *U* test was used. For categorical variables, Pearson’s chi-squared test was performed. Standardized mean differences (SMDs) are presented to describe the differences between cohorts with and without AKI.^20^

A logistic regression model was fitted to assess the relationship between AKI, country income level and in-hospital death. All variables known to indicate disease severity and increased susceptibility to AKI from Table 1 were initially selected. However, many of them were excluded because their sample size was too small, likely from under-reporting, leading to significant class imbalance.

**Table 1:** Characteristics of patients with AKI and no AKI grouped by country income level

Variables	Low and lower-middle income country patients				Upper-middle income country patients				High income country patients			
	All	AKI	No AKI	SMD	All	AKI	No AKI	SMD	All	AKI	No AKI	SMD
Total count	5238	2789	2449	---	1833	704	1129	---	25,049	7645	17,404	---
Demographics												
Age, y, median (IQR)	60 (50–70)	62 (52–71)	60 (49–70)	−0.13	58.1 (47–69)	63 (52–71)	55.1 (43.1–68)	−0.35	61 (51–70)	63 (54–70)	60 (50–69)	−0.19
Female (%)	1816 (35)	933 (33)	883 (36)	0.06	650 (35)	252 (36)	398 (35)	0.01	8172 (33)	2131 (28)	6041 (35)	0.15
AKI grades and KRT, n (%)												
AKI grade 1	916 (17)	916 (33)	0 (0)	---	145 (8)	145 (21)	0 (0)	---	1973 (8)	1973 (26)	0 (0)	---
AKI grade 2	463 (9)	463 (17)	0 (0)	---	73 (4)	73 (10)	0 (0)	---	841 (3)	841 (11)	0 (0)	---
AKI grade 3 with RRT	1410 (27)	1410 (51)^a^	0 (0)	---	486 (27)	486 (69)^a^	0 (0)	---	4831 (19)	4831 (63)^a^	0 (0)	---
AKI grade 3 w/o RRT		658 (24)^a^				169 (24)^a^				1414 (18)a^a^		
KRT	752 (14)	752 (27)^a^	0 (0)	---	317 (17)	317 (45)^a^	0 (0)	---	3417 (14)	3417 (45)^a^	0 (0)	---
KRT/all grade 3		53%				65%				71%		
		Calculated as a proportion of total AKI^a^				Calculated as a proportion of total AKI^a^				Calculated as a proportion of total AKI^a^		
Comorbidities^b^, n (%)												
Chronic kidney disease	138 (3)	100 (4)	38 (2)	0.13	139 (8)	105 (15)	34 (3)	0.45	2047 (8)	1112 (15)	935 (5)	0.31
Chronic cardiac disease	523 (10)	291 (10)	232 (9)	0.03	255 (14)	133 (19)	122 (11)	0.24	3873 (15)	1410 (18)	2463 (14)	0.11
Chronic pulmonary disease	168 (3)	87 (3)	81 (3)	0.01	102 (6)	51 (7)	51 (5)	0.13	2463 (10)	756 (10)	1707 (10)	0.00
Hypertension	2451 (47)	1360 (49)	1091 (45)	0.08	950 (52)	435 (62)	515 (46)	0.36	9681 (39)	3115 (41)	6566 (38)	0.19
Dementia	20 (0)	9 (0)	11 (0)	0.02	23 (1)	6 (1)	17 (2)	0.06	249 (1)	80 (1)	169 (1)	0.01
Liver disease	34 (1)	21 (1)	13 (1)	0.03	26 (1)	17 (2)	9 (1)	0.14	801 (3)	286 (4)	515 (3)	0.04
Malnutrition	16 (0)	5 (0)	11 (0)	0.05	26 (1)	14 (2)	12 (1)	0.08	238 (1)	89 (1)	149 (1)	0.03
Obesity	113 (2)	57 (2)	56 (2)	0.02	517 (28)	216 (31)	301 (27)	0.14	6131 (24)	1923 (25)	4208 (24)	0.03
Signs and symptoms on admission, n (%)												
Altered consciousness/confusion	137 (3)	85 (3)	52 (2)	0.06	115 (6)	49 (7)	66 (6)	0.12	2563 (10)	894 (12)	1669 (10)	0.10
Diarrhea	185 (4)	99 (4)	86 (4)	0.00	256 (14)	95 (13)	161 (14)	0.03	4981 (20)	1424 (19)	3557 (20)	0.01
Fever	3756 (72)	1994 (71)	1762 (72)	0.02	1135 (62)	396 (56)	739 (65)	0.01	17,996 (72)	5393 (71)	12,603 (72)	0.03
Vomiting/nausea	268 (5)	139 (5)	129 (5)	0.01	160 (9)	47 (7)	113 (10)	0.09	4045 (16)	1137 (15)	2908 (17)	0.02
Muscle aches/joint pain	386 (7)	189 (7)	197 (8)	0.05	476 (26)	145 (21)	331 (29)	0.14	5880 (23)	1617 (21)	4263 (24)	0.04
Headache	184 (4)	96 (3)	88 (4)	0.01	279 (15)	69 (10)	210 (19)	0.22	3116 (12)	810 (11)	2306 (13)	0.06
Cough	1686 (32)	859 (31)	827 (34)	0.07	1009 (55)	356 (51)	653 (58)	0.00	16,925 (68)	4956 (65)	11,969 (69)	0.02
Shortness of breath	3904 (75)	2155 (77)	1749 (71)	0.12	1,144 (62)	442 (63)	702 (62)	0.18	19,462 (78)	5773 (76)	13,689 (79)	0.01
Observations on admission, median (IQR)												
Temperature, C	36.7 (36.7–37)	36.7 (36.7–37)	36.7 (36.7–37.1)	0.10	36.8 (36.2–37.7)	37 (36.3–37.7)	36.8 (36.2–37.6)	−0.08	37.5 (36.8–38.3)	37.5 (36.8–38.4)	37.5 (36.8–38.3)	−0.04
Systolic BP, mmHg	130 (117–143)	130 (117–145)	130 (118–141)	−0.03	130 (116–144)	129 (111–145)	130 (119–143)	0.08	127 (112–142)	127 (110–144)	127 (112–142)	−0.01
Diastolic BP, mmHg	75 (68–83)	75 (66–84)	75 (69–82)	−0.02	76 (66–85)	74 (63–85)	77 (68–85)	0.18	73 (63–82)	71 (60–81)	73 (64–82)	0.14
Heart rate, BPM	91 (82–103)	94 (82.5–107)	90 (81–100)	−0.18	91 (80–106)	94 (80–108)	90 (79–104)	−0.21	94 (82–108)	95 (82–109)	94 (82–108)	−0.05
Respiratory rate, per min	24 (20–29)	24 (21–30)	23 (20–28)	−0.23	22 (18–28)	24 (20–30)	21 (18–26)	−0.38	24 (20–30)	24 (20–31)	24 (20–30)	−0.02
Oxygen saturation, %	93 (90–96)	92.4 (89–96)	94 (90–96)	0.15	94 (90–96)	92 (88–96)	94 (91–97)	0.32	93.3 (89–96)	93 (88–96)	94 (89.8–96)	0.14
Laboratory results on admission, median (IQR)												
Potassium (mmol/l)	4.2 (3.7–4.6)	4.2 (3.7–4.6)	4.1 (3.7–4.5)	−0.14	4.1 (3.7–4.5)	4.2 (3.8–4.6)	4.1 (3.7–4.5)	−0.24	4.1 (3.7–4.4)	4.2 (3.8–4.6)	4 (3.7–4.4)	−0.24
sCr (μmol/l)	88.4 (70.7–132.6)	97.2 (70.7–177.5)	84 (68.1–106.1)	−0.48	87.8 (67.4–117.6)	100.8 (71.5–150.5)	83.1 (66.1–102.6)	−0.48	84.9 (67.2–111)	98 (74.3–153)	80.4 (65–101)	−0.50
eGFR (ml/min per 1.73 m^2^)	73 (44.2–96.5)	64 (30.9–93.9)	80.4 (57.2–98.7)	0.45	78 (52.2–99.5)	61.6 (37–93.1)	84.3 (63.4–102.4)	0.58	80.7 (55.9–98)	66.3 (36.8–90.6)	85.3 (63.9–100.4)	0.59
Hemoglobin (g/l)	128 (111–141)	126 (109–140)	129 (115–142)	0.17	135 (123–148)	130 (116.2–145)	138 (126–150)	0.38	135 (120–147)	131 (114–145)	136 (122–148)	0.26
Sodium (mmol/l)	137 (134–141)	138 (134–142)	137 (133.8–141)	−0.10	137 (134.3–140)	137 (134–140)	137 (135–140)	0.01	136 (133–139)	136 (133–139)	136 (133–139)	0.01
Admission Treatment, n (%)												
Antiviral and COVID-19 targeting agents	2633 (50)	1464 (52)	1169 (48)	0.09	256 (14)	108 (15)	148 (13)	0.16	11,066 (44)	2663 (35)	8403 (48)	0.27
Antibiotic agents	4733 (90)	2640 (95)	2093 (85)	0.30	1399 (76)	506 (72)	893 (79)	0.34	22,631 (90)	7145 (93)	15,486 (89)	0.19
Antifungal agents	133 (3)	86 (3)	47 (2)	0.07	205 (11)	127 (18)	78 (7)	0.41	4119 (16)	1865 (24)	2254 (13)	0.34
Corticosteroids	3699 (71)	2183 (78)	1516 (62)	0.36	1180 (64)	455 (65)	725 (64)	0.19	12,018 (48)	3907 (51)	8111 (47)	0.10
Invasive mechanical ventilation	4851 (93)	2668 (96)	2183 (89)	0.25	1060 (58)	557 (79)	503 (45)	1.12	16,769 (67)	6582 (86)	10,187 (59)	0.65
Complications^b^, n (%)												
Bacterial pneumonia	192 (4)	120 (4)	72 (3)	−0.05	478 (26)	266 (38)	212 (19)	0.60	5582 (22)	2044 (27)	3538 (20)	0.19
Cardiac arrest	570 (11)	386 (14)	184 (8)	−0.15	192 (10)	140 (20)	52 (5)	0.61	1725 (7)	1048 (14)	677 (4)	0.37
Coagulation disorder	167 (3)	93 (3)	74 (3)	−0.01	79 (4)	68 (10)	11 (1)	0.49	2180 (9)	937 (12)	1243 (7)	0.21
Rhabdomyolysis	1 (0)	1 (0)	0 (0)	−0.02	16 (1)	12 (2)	4 (0)	0.17	429 (2)	224 (3)	205 (1)	0.14
Outcomes, n (%)												
Transferred	244 (5)	89 (3)	155 (6)	0.15	72 (4)	31 (4)	41 (4)	0.04	2542 (10)	925 (12)	1617 (9)	0.09
Discharged	1801 (34)	494 (18)	1307 (53)	0.80	1113 (61)	208 (30)	905 (80)	1.18	14,402 (57)	2591 (34)	11,811 (68)	0.72
Death	3193 (61)	2206 (79)	987 (40)	0.86	648 (35)	465 (66)	183 (16)	1.17	8105 (32)	4129 (54)	3976 (23)	0.68
Length of stay (median, IQR)	7 (4–11)	8 (5–13)	6 (4–9)	−0.36	14 (8–24)	18.5 (11–30)	12 (7–20)	−0.48	17 (10–28)	20 (11–33)	16 (10–25)	−0.26

AKI, acute kidney injury; BP, blood pressure; BPM, beats per minute; eGFR, estimated glomerular filtration rate (estimated using the CKD-EPI equation); IQR, interquartile range; RRT, renal replacement therapy; RRT, renal replacement therapy; sCr, serum creatinine; SMD, standardized mean difference.

a Calculated as a proportion of total AKI.

b Definitions of comorbidities, complications and outcomes from the CRFs are presented in Supplementary Table S2.

The final list of variables included age, sex and socioeconomic status, mechanical ventilation, and clinical observations on admission. Information pertaining to sex represents “sex at birth”. Continuous variables were binned into clinically meaningful categories to help account for nonlinear relationships with mortality. Several categories with few observations were subsequently removed. The reported model was fit to data from the entire inclusion cohort but held HIC as the reference group to specifically evaluate the association between being from a LLMIC with in-hospital death. Multiple imputation by chained equations was used to address variable missingness.

The relationship between AKI, no AKI and in-hospital death and discharge was described for each country income group with a survival curve approximated using a Kaplan–Meier estimator.^21^ The follow-up period, measured in days, began on the day of hospital admission and ended on the day of either discharge or death. Hospital discharge and transfer were considered absorbing states, exclusive with hospital morbidity.

All statistical analyses were performed using the R statistical programming language, version 4.1.2.^22^^,^^23^

## Results

**Figure 1:**
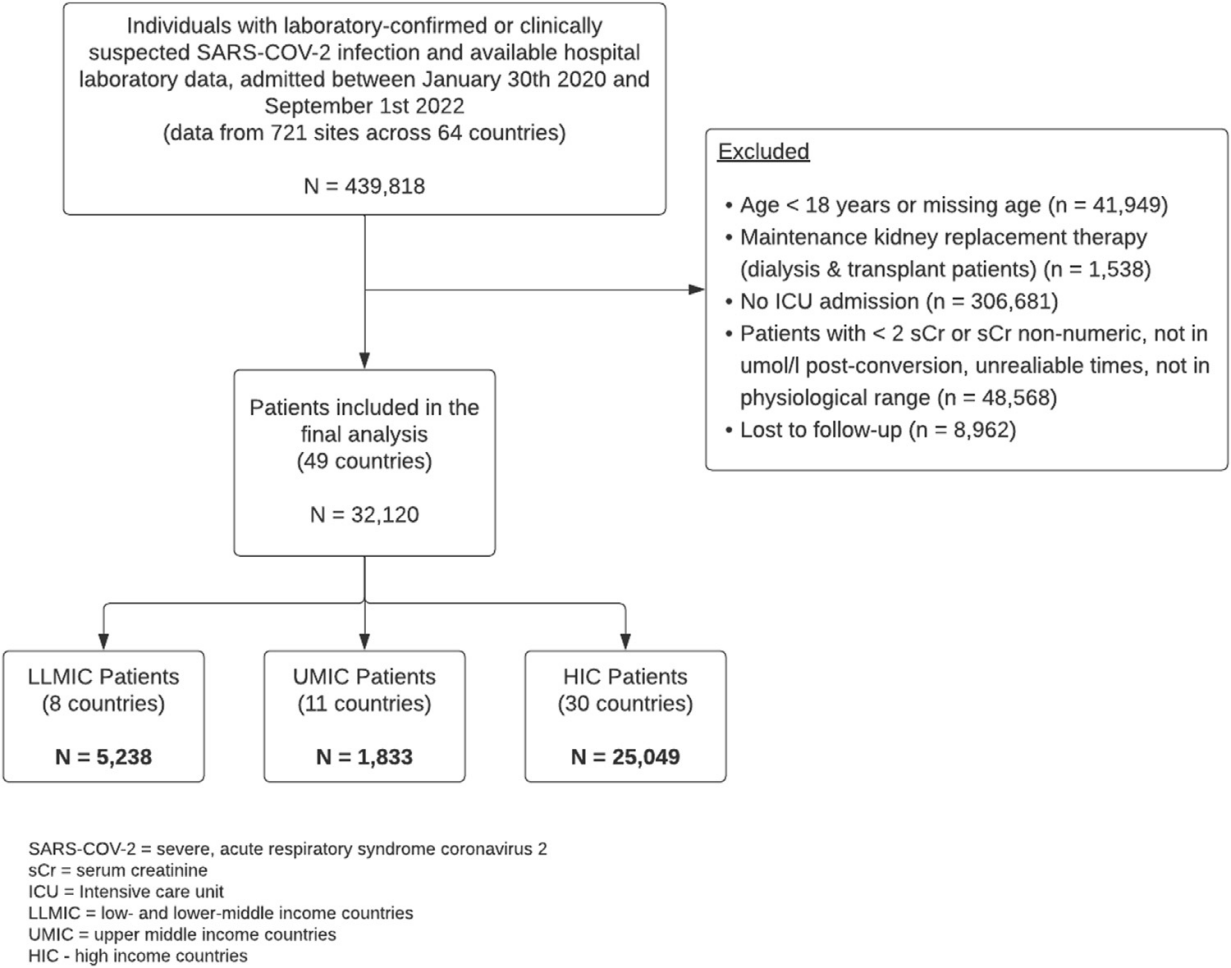
Flowchart of the study.

**Figure 2:**
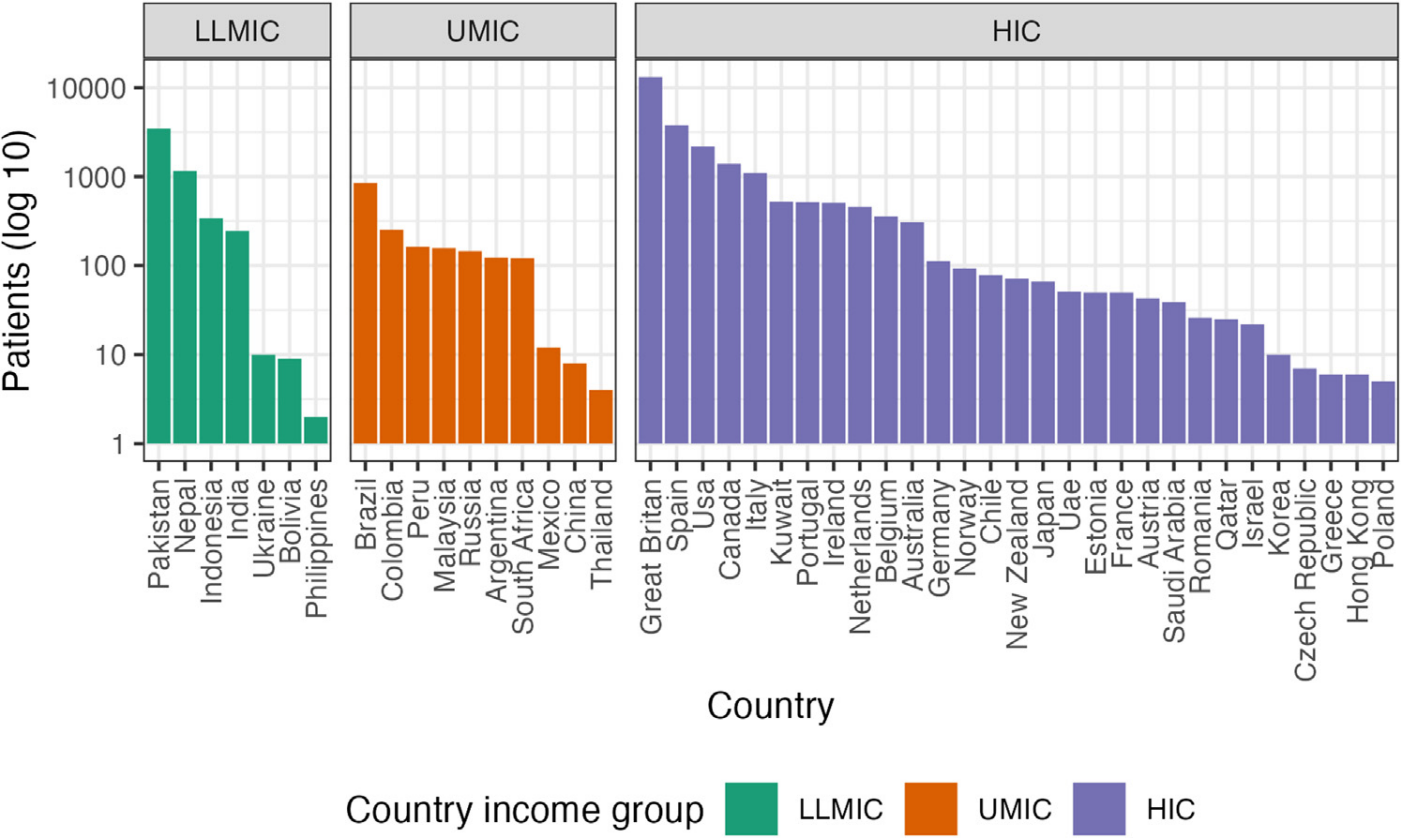
Analysis cohort contributing countries. HIC, high income country; LLMIC, low- and low-middle income country; UMIC, upper-middle income country. ∗ Countries that contributed only 1 patient are not presented in this figure.

**Figure 3:**
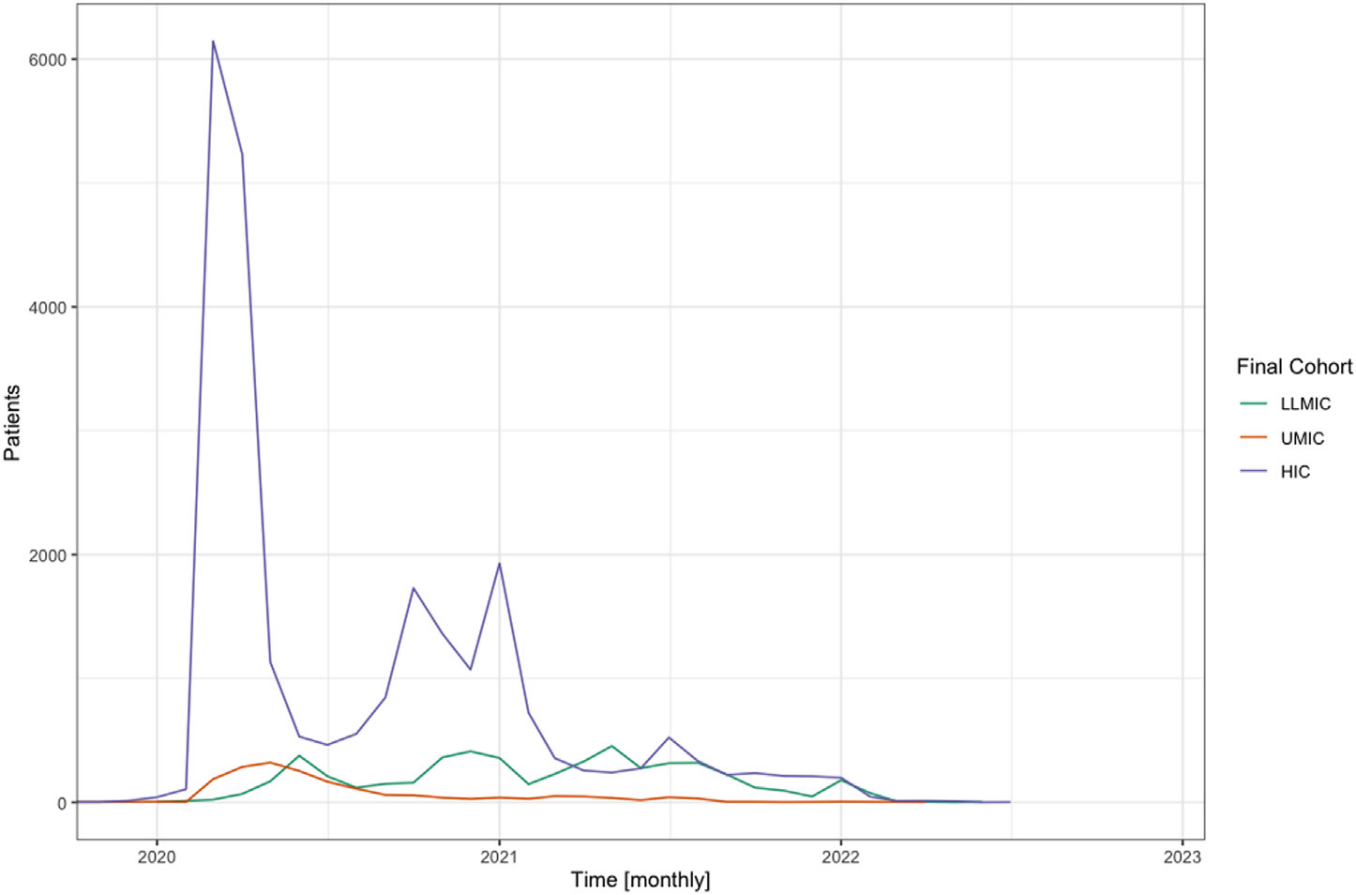
Temporal distribution of admissions by country income group. HIC, high income country; LLMIC, low- and low-middle income country; UMIC, upper-middle income country.

Data were collected for 439,818 individuals from 721 sites and 64 countries. Of these, 32,120 were used as the analysis cohort (Figure 1). A breakdown of the 2 main groups of excluded patients (ICU admission and <2 sCr) by country income level and individual country can be seen in Supplementary Figure S1. There were 5238 patients from 8 LLMICs, 1833 patients from 11 UMICs and 25,049 patients from 30 HICs (Figure 2). The LLMIC group was mainly constituted by patients from various regions in Asia, whereas the UMIC group had a predominance of patients from Latin America, and the HIC group from North America and Western Europe. Admissions peaked in the first half of 2020 for HIC patients and then increased again at the end of that year and in early 2021. Patients from LLMIC were mainly admitted through the first 8 months of 2020 whereas UMIC were more evenly admitted from late 2020 to the second half of 2021 (Figure 3).

The median length of stay was 7 days (IQR 4–11) for LMIC patients, 14 days (IQR 8–24) for UMIC patients, and 17 days (IQR 10–28) for HIC patients (Table 1). Missing data were highly variable between country income groups (Supplementary Table S4).

### Incidence, Staging, and Timing of Peak AKI

**Figure 4:**
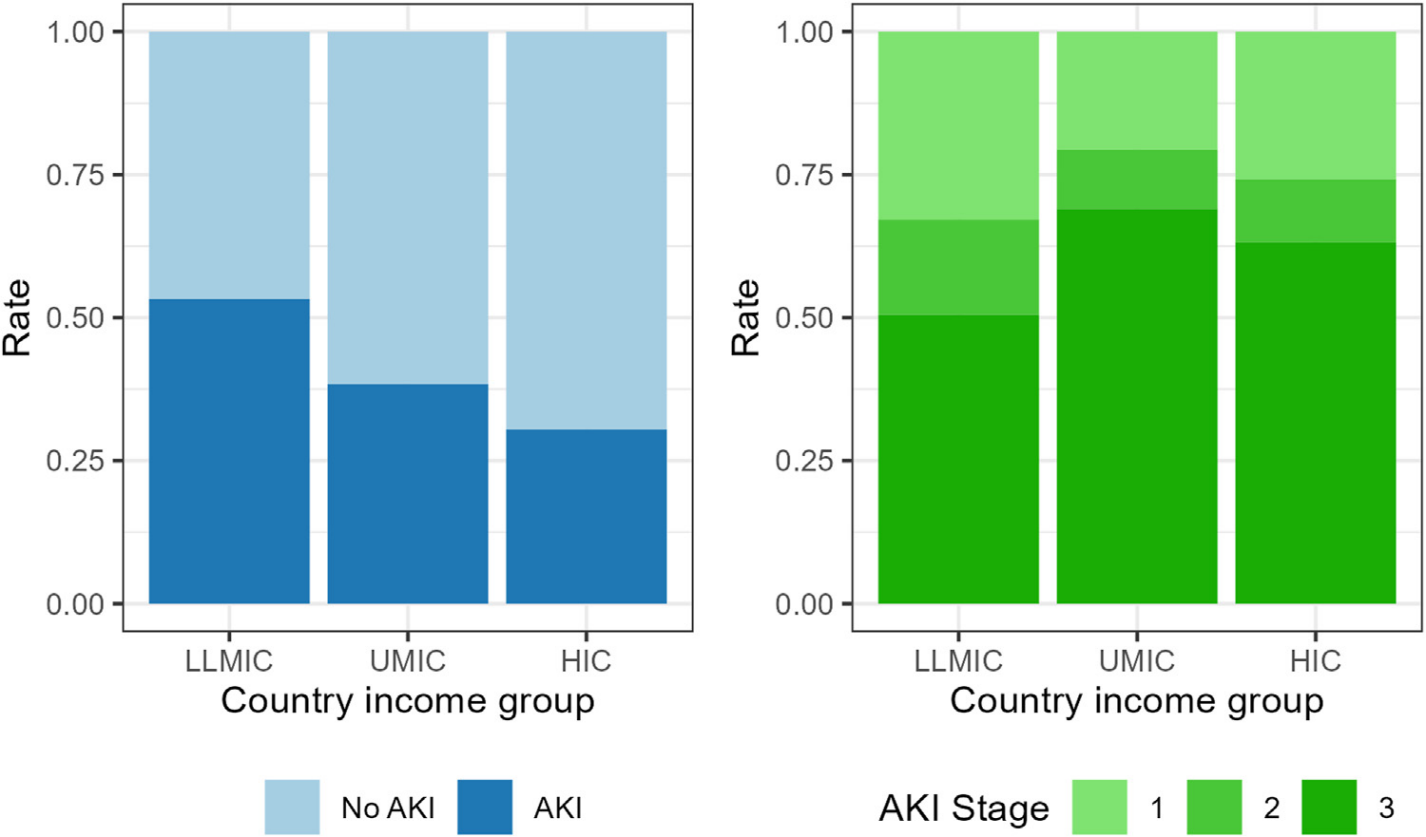
AKI incidence and breakdown by country income group. AKI, acute kidney injury; HIC, high income country; LLMIC, low- and low-middle income country; UMIC, upper-middle income country.

**Figure 5:**
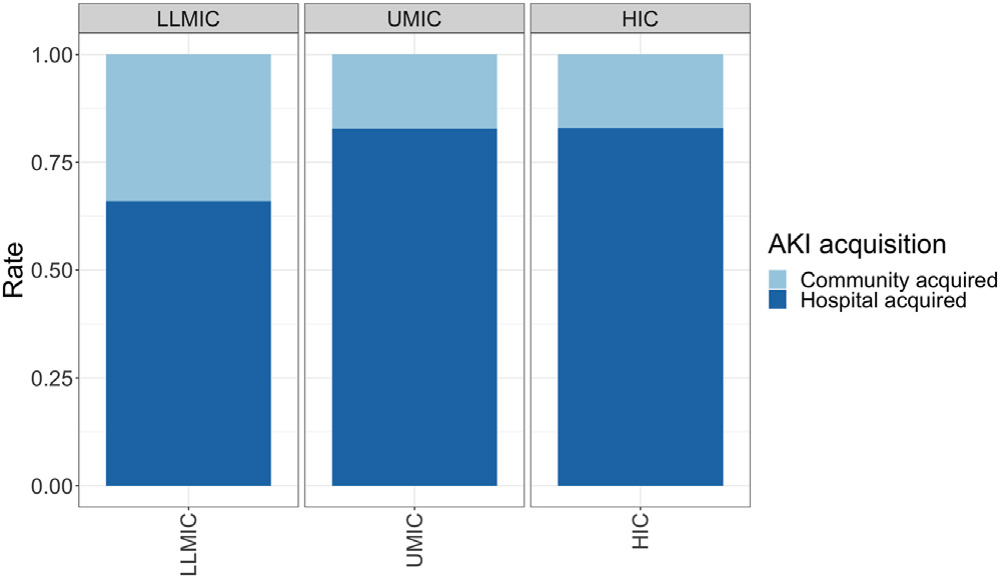
Community-acquired versus hospital-acquired acute kidney injury based on 48 hour cutoff from admission by country income group. AKI, acute kidney injury; HIC, high income country; LLMIC, low- and low-middle income country; UMIC, upper-middle income country.

Among patients with COVID-19 admitted to the ICU, AKI incidence was highest in LLMIC, followed by UMIC and HIC patients (53%, 38% and 30%, respectively) (Figure 4 and Supplementary Figure S2 for individual country breakdown). Among all patients with AKI, those from UMIC had the highest proportion of stage 3 AKI (69%), followed by HIC (63%) and LLMIC (51%) (Figure 4). Patients from HIC with AKI had the highest rate of acute dialysis treatment overall (45%) and as a proportion of all patients with stage 3 AKI (71%) (Table 1). The lowest rate of dialysis overall and as a proportion of stage 3 AKI was seen in LLMIC patients (27% and 53%, respectively) with UMIC falling in the middle. The rates of AKI stage 3 without dialysis were similar between the 3 groups, with the highest observed in LLMIC and UMIC (24%) and lowest in HIC (18%). Peak sCr occurred more frequently on days 1 to 3 of admission in LLMIC patients and decreased sequentially thereafter, whereas for UMIC and HIC, peak AKI was spread variably across admission days (Supplementary Figure S3). Whereas hospital-acquired AKI was the predominant type of AKI, LLMIC patients had the largest proportion of CA-AKI (34% vs. 17% in HIC and UMIC) (Figure 5 and Supplementary Figure S4 for individual country breakdown)

### Demographic and Clinical Characteristics

Baseline characteristics at hospital admission, acute interventions, complications, and outcomes for patients with AKI versus no-AKI for each country income group are provided in Table 1. Median age was between 58 and 61 years across the 3 groups and similar proportions, approximately one-third, were female. With the exception of hypertension, the prevalence of all comorbidities among LLMIC patients, particularly with regards to chronic kidney disease, appeared significantly underreported considering known global estimates.^24^ Hypertension was highly prevalent in all patient groups, especially among patients with AKI who are from LLMIC (49%) and UMIC (62%) whereas approximately one-quarter of UMIC and HIC patients (28% and 24%, respectively) were obese. Shortness of breath, cough, and fever were the most common presenting symptoms on admission in all patients.

Kidney function on admission in patients with and without AKI was comparable between country income groups. However, a significant improvement in estimated glomerular filtration rate (estimated with CKD-EPI equation and no “if black” correction), and concurrent drop in sCr, between patients with AKI and those without AKI could be seen in all income groups (median estimated glomerular filtration rates, LLMIC: 64 vs. 80 ml/min per 1.73 m^2^, UMIC: 62 vs. 84 ml/min per 1.73 m^2^, HIC: 66 vs. 85 ml/min per 1.73 m^2^) (SMD 0.45 to 0.59).

Patients with AKI from LLMIC were the most likely to be treated with antivirals and COVID-19 related medications (52%), antibiotics (95%), corticosteroids (78%), and be supported with invasive mechanical ventilation (96%) (Supplementary Figure S5).

### Outcomes

**Figure 6:**
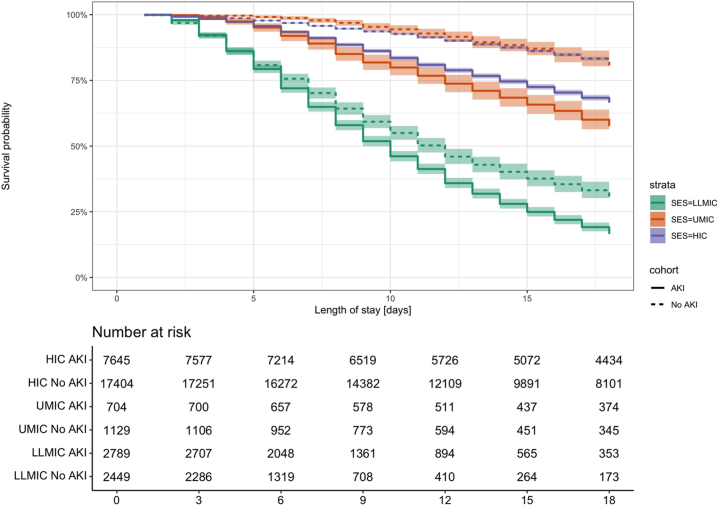
Kaplan–Meier Survival plot stratified across acute kidney injury and country income groups. Confidence bars are used to illustrate a 95% confidence interval. AKI, acute kidney injury; HIC, high income country; LLMIC, low- and low-middle income country; SES, socioeconomic status; UMIC, upper-middle income country.

In-hospital death was highest among patients from LLMIC (61%) with a significant difference observed between patients who developed AKI (79%) and those who did not (40%) (*P* < 0.001, SMD 0.80). Approximately one-third (32%) of all patients from HIC died, and this proportion increased to 54% among patients with AKI. Interestingly, this difference in mortality based on AKI status was most pronounced in patients from UMIC among whom 66% of those with AKI but only 16% of those without AKI died, making the mortality of patients without AKI from UMIC and HIC comparable (Figure 6).

After adjusting for markers of disease severity and AKI susceptibility, the risk of in-hospital death with AKI persisted and increased with AKI severity (Table 2). Being from LLMIC (vs. HIC) was associated with an increased risk of death; however, this was not the case for patients from UMIC (vs. HIC). Other factors that were associated with a significantly higher risk of death included age over 65 years and treatment with antibiotics.

**Table 2:** Logistic regression fitted to assess the association between AKI and in-hospital death

Variables	Odds ratio	95% confidence interval		*P*-value
		Lower	Upper	
(intercept)	0.109	0.099	0.120	< 0.001
AKI grade 1	2.026	1.863	2.203	< 0.001
AKI grade 2	4.290	3.796	4.852	< 0.001
AKI grade 3	5.230	4.903	5.579	< 0.001
SES: LLMIC	2.699	2.509	2.902	< 0.001
SES: UMIC	1.066	0.951	1.194	0.271
Age 65+^a^	2.838	2.686	2.998	< 0.001
Female	0.895	0.847	0.945	< 0.001
Hypertension	0.944	0.895	0.996	0.035
Temperature [0, 36]	0.935	0.825	1.059	0.292
Temperature [38+]	0.940	0.885	0.998	0.042
Systolic BP [0, 100]	1.126	1.027	1.233	0.011
Systolic BP [140+]	0.996	0.941	1.055	0.9
Heart rate [0, 60]	0.877	0.743	1.033	0.12
Heart rate [100+]	1.129	1.069	1.193	< 0.001
Respiratory rate [0, 12]	1.237	0.837	1.817	0.281
Respiratory rate [20+]	1.047	0.977	1.122	0.193
Oxygen saturation [0, 90]	1.600	1.499	1.708	< 0.001
Oxygen saturation [90 - 95]	1.088	1.0231	1.156	0.007
Mechanical ventilation	1.690	1.586	1.801	< 0.001

AKI, acute kidney injury; BP, blood pressure; LLMIC, low- and low-middle income country; MiCE imputation used for variable missingness; ref, reference; SES, socioeconomic status; UMIC, upper-middle income country.

Multiple imputation by chained equations.

a Reference for age = 18 - 65 years old.

## Discussion

In the first and largest study of the association of country income level with the characteristics and outcomes of hospitalized patients with AKI and COVID-19, we observed that ICU patients from lower income countries were more likely to develop AKI, more likely to acquire AKI in the community, less likely to receive acute dialysis, and approximately twice as likely to die than patients from wealthier countries, with or without AKI.

The incidence of AKI in COVID-19 cohort studies since 2020 has been enormously variable and subject to differences in the proportions of critically ill patients, the definitions used to diagnose AKI, the levels of resourcing to detect AKI by individual sites, and the predominant COVID variant at the time.^25^ Differences in AKI incidence must therefore be examined and interpreted within this context of variability. Our reported incidences of AKI among patients from HIC, UMIC, and LLMIC are consistent with the current limited available literature. The HIC incidence of 30% is similar to a Belgian multicenter study that examined an ICU-only cohort.^26^ The UMIC AKI incidence of 38% is comparable to that of 2 separate studies from general hospital patients in Mexico and 1 study from 2 tertiary centers in South Africa (30%–34%).^12^^,^^27^^,^^28^ The LLMIC incidence (53%), has also been reported in small cohorts of both critical and noncritical hospitalized patients from India and Pakistan.^29^^,^^30^ Higher incidences of AKI (up to 85%) have been reported in HIC ICU patient studies that include the urine output component of the Kidney Disease Improving Global Outcomes definition, suggesting that combining both elements of the definition captures a far broader class of patients than considered here.^26^^,^^31^

Whereas patients from LLMIC had a higher proportion of CA-AKI (∼30%) using the 48 hour threshold definition, hospital-acquired AKI remained the predominant form of AKI in all groups. A registry wide study of AKI in COVID-19 conducted by the Sociedad Latinoamericana de Nefrologia e Hipertension of 872 patients from 12 countries in the region, similarly found that 64% of patients with AKI had hospital-acquired AKI.^32^ The higher proportion of CA-AKI in LLMIC, than in UMIC and HIC, agrees with the global AKI literature and likely reflects limitations in access to health care, particularly in the community, as well as a greater reluctance to seek medical attention leading to delayed presentations.^9^^,^^12^^,^^33^

The lower incidence of acute dialysis in our LLMIC population (27% vs. 45% in HIC and UMIC) is likely due to multiple factors. A possible explanation is that the limited dialysis capacity in LLMIC sites meant that patients with AKI stage 3 and borderline indications for dialysis were more likely to be treated conservatively, at least initially. The recommendations for dialysis in COVID-19 have been the same as those set forth by general guidelines, with no particular indication toward earlier starts or bypassing of diuretic challenges in fluid overloaded patients.^34^ Alternatively, the observed differences in dialysis rates between socioeconomic groups in our study may result squarely from inequities in kidney care capacity and delivery. Resource shortage leading to selective treatment allocation has been a critical challenge during this pandemic, especially among health facilities in low-income and middle-income countries.^35^ Acute dialysis, usually in the form of continuous hemodialysis, is not only costly but requires highly trained staff to support it. The ISN dialysis outcomes and practice patterns survey described major supply disruptions in hemodialysis consumables like dialyzers and dialysate solutions as well as disruptions to hemodialysis water processing and more frequent reduction in dialysis session length in LLMIC.^36^ In the Sociedad Latinoamericana de Nefrologia e Hipertension study, 43 patients (4.9%) had indications for dialysis but did not receive it, presumably because of supply shortages, and 18% of nephrologists surveyed stated that they were not able to provide dialysis because of limited resource allocation.^32^ Although it is not possible to know whether this is what led to the observed differences across income groups, the global inequities in kidney care have been well documented over time and demand greater action from ruling national authorities as well as the nephrology community at large.^37^

Although it is known that AKI is associated with an increased risk of death in COVID-19, our finding that this risk almost doubled when shifting from an HIC to an LLMIC population is a powerful reminder of the impact of socioeconomic circumstance. Susantitaphong *et al.* clearly illustrate this inverse relationship between AKI-associated mortality and gross national income per capita suggesting there may be significant, but often underreported, differences in health care delivery that account for these findings.^10^ At present, of the 12 nations with the highest burden of COVID-19 related deaths, 8 are middle or low-income countries.^38^ The infection fatality rate is estimated to be 2.7 times higher in the general population and up to 5.4 times higher among people between the ages of 60 and 80 years in these countries.^8^^,^^38^ In this context, our finding of income level, particularly LLMIC, being an independent predictor of death is not surprising. Similarly, the ISN dialysis outcomes and practice patterns survey of dialysis units around the world, revealed an excess mortality from COVID-19 in patients from LLMIC on maintenance hemodialysis of over 50%.^36^ Although COVID-19 served to expose the fragility in the healthcare infrastructure of these countries, factors such as the ability to isolate within one’s own home and to work remotely without facing financial hardship became key drivers of infection in these regions.^39^ Furthermore, the rise in vaccine nationalism among wealthy nations meant that those with the highest burden of infection and limited capacity to isolate or access quality healthcare, were the last to be vaccinated and the first to die.^40^

There are some key limitations in our study. The ISARIC data collection was a voluntary assembly of data generated during an evolving pandemic. The representation of individual countries or regions was therefore conditioned by the capacity and resources of healthcare workers and institutions to collect and process data, leading to sampling bias and limiting comparisons to global estimates. Considering that this was an ICU-only study, treatment and outcome differences will inevitably reflect differences in the ICU admission criteria of each country during the pandemic. With limited resources and large case numbers, it is likely that ICUs in LLMICs had to apply a higher threshold for admission, thereby selecting a more critically ill population. This would explain why a greater proportion of these patients were treated with mechanical ventilation, corticosteroids, and antivirals when compared with those in higher income country ICUs. Nevertheless, as the largest and most diverse international data set of hospitalized COVID-19 patients, our study provides invaluable insights into the impact of this outbreak on people from widely variable demographic, cultural, and socioeconomic backgrounds.^41^ In our study, the exclusion of patients without an ICU admission or 2 sCr measurements may have introduced a degree of selection bias and the lack of a time-standardized collection of sCr across all sites also represents a limitation of the study. The exclusion of patients lost to follow-up may have also introduced further bias. Finally, the predominant representation of patients from Asian countries in the LLMIC group and the fact that these sites collected data from ICU patients only, means that generalizability to other LLMIC regions and settings is uncertain.

To our knowledge, this is the first study to systematically examine the association of country income level with the characteristics and outcomes of patients with AKI and COVID-19. Our population is, as far as we know, the largest and only multinational cohort of patients with COVID-19 from all country income levels with data extending to the latter part of 2022. Patients from poorer countries had more AKI, received less dialysis, and died at twice the rate of patients from wealthier countries. Experiencing an episode of AKI and being from an LMIC country were independent predictors of mortality, suggesting that AKI is a particularly devastating complication of COVID-19 in patients from poorer nations, especially when admitted to an ICU, where the gaps in accessibility and quality of healthcare delivery have a major impact on patient outcomes.

## Disclosure

MW declared funding from the University of Queensland’s Research and Training Scholarship and the Digital Health CRC of Australia. NS and SS declared funding from Artificial Intelligence for Pandemics (A14PAN) at University of Queensland. MG declared funding from the University of Queensland. SS declared funding from The Australian Research Council Centre of Excellence for Engineered Quantum Systems (EQUS, CE170100009). LM declared funding from UK Foreign, Commonwealth and Development Office and Wellcome [215091/Z/18/Z], Bill & Melinda Gates Foundation [OPP1209135]. DJ declared funding from research grants from Baxter and Fresenius Medical Care and the Australian National Health and Medical Research. All other authors declared no specific funding for this work. The funders had no role in study design, data collection and analysis, decision to publish, or preparation of the manuscript.

## Appendix

### Members of the ISARIC Clinical Characterization Group

Sheryl Ann Abdukahil; Nurul Najmee Abdulkadir; Ryuzo Abe; Laurent Abel; Amal Abrous; Lara Absil; Andrew Acker; Shingo Adachi; Diana Adrião; Saleh Al Ageel; Shakeel Ahmed; Kate Ainscough; Eka Airlangga; Tharwat Aisa; Ali Ait Hssain; Younes Ait Tamlihat; Takako Akimoto; Ernita Akmal; Chika Akwani; Eman Al Qasim; Razi Alalqam; Angela Alberti; Tala Al-dabbous; Senthilkumar Alegesan; Marta Alessi; Beatrice Alex; Kévin Alexandre; Abdulrahman Al-Fares; Huda Alfoudri; Imran Ali; Kazali Enagnon Alidjnou; Jeffrey Aliudin; Qabas Alkhafajee; Clotilde Allavena; Nathalie Allou; João Alves; Rita Alves; João Melo Alves; Joana Alves Cabrita; Maria Amaral; Nur Amira; Phoebe Ampaw; Roberto Andini; Claire Andréjak; Andrea Angheben; François Angoulvant; Séverine Ansart; Sivanesen Anthonidass; Massimo Antonelli; Carlos Alexandre Antunes de Brito; Ardiyan Apriyana; Yaseen Arabi; Irene Aragao; Francisco Arancibia; Carolline Araujo; Antonio Arcadipane; Patrick Archambault; Lukas Arenz; Jean-Benoît Arlet; Christel Arnold-Day; Lovkesh Arora; Rakesh Arora; Elise Artaud-Macari; Diptesh Aryal; Angel Asensio; Muhammad Ashraf; Jean Baptiste Assie; Amirul Asyraf; Minahel Atif; Anika Atique; Johann Auchabie; Hugues Aumaitre; Adrien Auvet; Laurène Azemar; Cecile Azoulay; Benjamin Bach; Delphine Bachelet; Claudine Badr; J. Kenneth Baillie; J Kevin Baird; Erica Bak; Agamemnon Bakakos; Nazreen Abu Bakar; Andriy Bal; Mohanaprasanth Balakrishnan; Valeria Balan; Firouzé Bani-Sadr; Renata Barbalho; Nicholas Yuri Barbosa; Wendy S. Barclay; Saef Umar Barnett; Michaela Barnikel; Audrey Barrelet; Cleide Barrigoto; Marie Bartoli; Joaquín Baruch; Romain Basmaci; Muhammad Fadhli Hassin Basri; Denise Battaglini; Jules Bauer; Diego Fernando Bautista Rincon; Abigail Beane; Alexandra Bedossa; Ker Hong Bee; Husna Begum; Sylvie Behilill; Albertus Beishuizen; Aleksandr Beljantsev; David Bellemare; Anna Beltrame; Beatriz Amorim Beltrão; Marine Beluze; Nicolas Benech; Lionel Eric Benjiman; Dehbia Benkerrou; Suzanne Bennett; Binny Benny; Luís Bento; Jan-Erik Berdal; Delphine Bergeaud; Hazel Bergin; José Luis Bernal Sobrino; Giulia Bertoli; Lorenzo Bertolino; Simon Bessis; Sybille Bevilcaqua; Karine Bezulier; Amar Bhatt; Krishna Bhavsar; Claudia Bianco; Farah Nadiah Bidin; Moirangthem Bikram Singh; Felwa Bin Humaid; Mohd Nazlin Bin Kamarudin; Zeno Bisoffi; François Bissuel; Patrick Biston; Laurent Bitker; Jonathan Bitton; Pablo Blanco-Schweizer; Catherine Blier; Frank Bloos; Mathieu Blot; Lucille Blumberg; Filomena Boccia; Laetitia Bodenes; Debby Bogaert; Anne-Hélène Boivin; Isabela Bolaños; Pierre-Adrien Bolze; François Bompart; Diogo Borges; Raphaël Borie; Elisabeth Botelho-Nevers; Lila Bouadma; Olivier Bouchaud; Sabelline Bouchez; Dounia Bouhmani; Damien Bouhour; Kévin Bouiller; Laurence Bouillet; Camile Bouisse; Anne-Sophie Boureau; John Bourke; Maude Bouscambert; Aurore Bousquet; Jason Bouziotis; Bianca Boxma; Marielle Boyer-Besseyre; Maria Boylan; Fernando Augusto Bozza; Axelle Braconnier; Cynthia Braga; Filipa Brás Monteiro; Luca Brazzi; Patrick Breen; Dorothy Breen; David Brewster; Kathy Brickell; Shaunagh Browne; Marjolein Brusse-Keizer; Petra Bryda; Nina Buchtele; Polina Bugaeva; Marielle Buisson; Erlina Burhan; Aidan Burrell; Ingrid G. Bustos; Denis Butnaru; André Cabie; Susana Cabral; Eder Caceres; Cyril Cadoz; Rui Caetano Garcês; Kate Calligy; Jose Andres Calvache; João Camões; Valentine Campana; Paul Campbell; Josie Campisi; Mireia Cantero; Pauline Caraux-Paz; Chiara Simona Cardellino; Sofia Cardoso; Filipe Cardoso; Filipa Cardoso; Nelson Cardoso; Simone Carelli; Nicolas Carlier; Thierry Carmoi; Gayle Carney; Inês Carqueja; Marie-Christine Carret; François Martin Carrier; Ida Carroll; Gail Carson; Maire-Laure Casanova; Mariana Cascão; Siobhan Casey; José Casimiro; Bailey Cassandra; Nidyanara Castanheira; Guylaine Castor-Alexandre; Ivo Castro; Ana Catarino; François-Xavier Catherine; Paolo Cattaneo; Roberta Cavalin; Alexandros Cavayas; Minerva Cervantes-Gonzalez; Anissa Chair; Catherine Chakveatze; Adrienne Chan; Meera Chand; Christelle Chantalat Auger; Jean-Marc Chapplain; Charlotte Charpentier; Julie Chas; Jonathan Samuel Chávez Iñiguez; Anjellica Chen; Yih-Sharng Chen; Léo Chenard; Matthew Pellan Cheng; Antoine Cheret; Thibault Chiarabini; Julian Chica; Suresh Kumar Chidambaram; Leong Chin Tho; Catherine Chirouze; Davide Chiumello; Sung-Min Cho; Bernard Cholley; Marie-Charlotte Chopin; Ting Soo Chow; Yock Ping Chow; Hiu Jian Chua; Jonathan Chua; Jose Pedro Cidade; Barbara Wanjiru Citarella; Anna Ciullo; Jennifer Clarke; Rolando Claure-Del Granado; Sara Clohisey; Perren J. Cobb; Cassidy Codan; Caitriona Cody; Alexandra Coelho; Megan Coles; Jennifer Coles; Gwenhaël Colin; Michael Collins; Sebastiano Maria Colombo; Pamela Combs; Jennifer Connolly; Marie Connor; Anne Conrad; Elaine Conway; Graham S. Cooke; Mary Copland; Hugues Cordel; Amanda Corley; Sabine Cornelis; Alexander Daniel Cornet; Arianne Joy Corpuz; Andrea Cortegiani; Grégory Corvaisier; Camille Couffignal; Sandrine Couffin-Cadiergues; Roxane Courtois; Stéphanie Cousse; Sabine Croonen; Gloria Crowl; Jonathan Crump; Claudina Cruz; Juan Luis Cruz Bermúdez; Jaime Cruz Rojo; Marc Csete; Ailbhe Cullen; Gerard Curley; Elodie Curlier; Colleen Curran; Paula Custodio; Ana da Silva Filipe; Charlene Da Silveira; Al-Awwab Dabaliz; Danyang Dai; Andrew Dagens; Darren Dahly; Heidi Dalton; Jo Dalton; Seamus Daly; Juliana Damas; Nick Daneman; Corinne Daniel; Emmanuelle A Dankwa; Jorge Dantas; Gillian de Loughry; Etienne De Montmollin; Rafael Freitas de Oliveira França; Rosanna De Rosa; Thushan de Silva; Peter de Vries; David Dean; Alexa Debard; Bianca DeBenedictis; Marie-Pierre Debray; Nathalie DeCastro; William Dechert; Lauren Deconninck; Romain Decours; Eve Defous; Isabelle Delacroix; Eric Delaveuve; Karen Delavigne; Andrea Dell'Amore; Christelle Delmas; Pierre Delobel; Corine Delsing; Elisa Demonchy; Emmanuelle Denis; Dominique Deplanque; Pieter Depuydt; Mehul Desai; Diane Descamps; Mathilde Desvallées; Santi Dewayanti; Pathik Dhanger; Alpha Diallo; Sylvain Diamantis; Fernanda Dias Da Silva; Juan Jose Diaz; Rodrigo Diaz; Kévin Didier; Jean-Luc Diehl; Wim Dieperink; Jérôme Dimet; Vincent Dinot; Fara Diop; Alphonsine Diouf; Yael Dishon; Félix Djossou; Annemarie B. Docherty; Helen Doherty; Maria Donnelly; Christl A. Donnelly; Sean Donohue; Yoann Donohue; Chloe Donohue; Peter Doran; Céline Dorival; Eric D'Ortenzio; James Joshua Douglas; Nathalie Dournon; Triona Downer; Joanne Downey; Mark Downing; Tom Drake; Aoife Driscoll; Murray Dryden; Claudio Duarte Fonseca; Vincent Dubee; François Dubos; Alexandre Ducancelle; Toni Duculan; Susanne Dudman; Abhijit Duggal; Paul Dunand; Jake Dunning; Mathilde Duplaix; Emanuele Durante-Mangoni; Lucian Durham III; Bertrand Dussol; Juliette Duthoit; Xavier Duval; Anne Margarita Dyrhol-Riise; Sim Choon Ean; Marco Echeverria-Villalobos; Eleanor Edwards; Siobhan Egan; Mohammed El Sanharawi; Subbarao Elapavaluru; Brigitte Elharrar; Philippine Eloy; Tarek Elshazly; Isabelle Enderle; Tomoyuki Endo; Chan Chee Eng; Ilka Engelmann; Vincent Enouf; Olivier Epaulard; Martina Escher; Mariano Esperatti; Hélène Esperou; Catarina Espírito Santo; Marina Esposito-Farese; João Estevão; Manuel Etienne; Nadia Ettalhaoui; Anna Greti Everding; Mirjam Evers; Marc Fabre; Isabelle Fabre; Amna Faheem; Arabella Fahy; Cameron J. Fairfield; Pedro Faria; Hanan Fateena; Arie Zainul Fatoni; Karine Faure; Raphaël Favory; Mohamed Fayed; Niamh Feely; Laura Feeney; Jorge Fernandes; Marília Andreia Fernandes; Susana Fernandes; François-Xavier Ferrand; Eglantine Ferrand Devouge; Joana Ferrão; Mário Ferraz; Sílvia Ferreira; Bernardo Ferreira; Benigno Ferreira; Nicolas Ferriere; Céline Ficko; Claudia Figueiredo-Mello; Juan Fiorda; Thomas Flament; Emily Flanagan; Clara Flateau; Tom Fletcher; Aline-Marie Florence; Letizia Lucia Florio; Deirdre Flynn; Jean Foley; Victor Fomin; Tatiana Fonseca; Simon Forsyth; Denise Foster; Giuseppe Foti; Erwan Fourn; Robert A. Fowler; Marianne Fraher; Diego Franch-Llasat; John F Fraser; Christophe Fraser; Marcela Vieira Freire; Ana Freitas Ribeiro; Caren Friedrich; Ricardo Fritz; Stéphanie Fry; Nora Fuentes; Masahiro Fukuda; Argin G; Valérie Gaborieau; Rostane Gaci; Massimo Gagliardi; Jean-Charles Gagnard; Amandine Gagneux-Brunon; Sérgio Gaião; Linda Gail Skeie; Phil Gallagher; Carrol Gamble; Yasmin Gani; Arthur Garan; Rebekha Garcia; Noelia García Barrio; Esteban Garcia-Gallo; Denis Garot; Valérie Garrait; Nathalie Gault; Aisling Gavin; Anatoliy Gavrylov; Alexandre Gaymard; Johannes Gebauer; Eva Geraud; Louis Gerbaud Morlaes; Nuno Germano; Jade Ghosn; Marco Giani; Jess Gibson; Tristan Gigante; Morgane Gilg; Elaine Gilroy; Guillermo Giordano; Michelle Girvan; Valérie Gissot; Daniel Glikman; Petr Glybochko; Eric Gnall; François Goehringer; Jean-Christophe Goffard; Jin Yi Goh; Jonathan Golob; Marie Gominet; Alicia Gonzalez; Patricia Gordon; Isabelle Gorenne; Conor Gormley; Laure Goubert; Cécile Goujard; Tiphaine Goulenok; Margarite Grable; Edward Wilson Grandin; Pascal Granier; Giacomo Grasselli; Christopher A. Green; William Greenhalf; Segolène Greffe; Domenico Luca Grieco; Matthew Griffee; Fiona Griffiths; Ioana Grigoras; Albert Groenendijk; Heidi Gruner; Yusing Gu; Jérémie Guedj; Martin Guego; Dewi Guellec; Daniela Guerreiro; Romain Guery; Anne Guillaumot; Laurent Guilleminault; Maisa Guimarães de Castro; Thomas Guimard; Marieke Haalboom; Daniel Haber; Hannah Habraken; Ali Hachemi; Nadir Hadri; Sheeba Hakak; Adam Hall; Matthew Hall; Sophie Halpin; Ansley Hamer; Raph L. Hamers; Rebecca Hamidfar; Terese Hammond; Naomi Hammond; Lim Yuen Han; Kok Wei Hao; Hayley Hardwick; Ewen M. Harrison; Janet Harrison; Alan Hartman; Junaid Hashmi; Ailbhe Hayes; Leanne Hays; Jan Heerman; Lars Heggelund; Ross Hendry; Martina Hennessy; Maxime Hentzien; Diana Hernandez; Andrew Hershey; Liv Hesstvedt; Eibhlin Higgins; Dawn Higgins; Rupert Higgins; Rita Hinchion; Samuel Hinton; Hiroaki Hiraiwa; Hikombo Hitoto; Antonia Ho; Yi Bin Ho; Alexandre Hoctin; Isabelle Hoffmann; Wei Han Hoh; Oscar Hoiting; Rebecca Holt; Jan Cato Holter; Peter Horby; Kota Hoshino; Ikram Houas; Catherine L. Hough; Stuart Houltham; Jimmy Ming-Yang Hsu; Jean-Sébastien Hulot; Abby Hurd; Samreen Ijaz; M. Arfan Ikram; Hajnal-Gabriela Illes; Patrick Imbert; Hugo Inácio; Yun Sii Ing; Mariachiara Ippolito; Sarah Isgett; Tiago Isidoro; Nadiah Ismail; Margaux Isnard; Junji Itai; Daniel Ivulich; Danielle Jaafar; Salma Jaafoura; Julien Jabot; Clare Jackson; Pierre Jaquet; Waasila Jassat; Coline Jaud-Fischer; Stéphane Jaureguiberry; Florence Jego; Anilawati Mat Jelani; Synne Jenum; Ong Yiaw Joe; Ruth N. Jorge García; Mark Joseph; Cédric Joseph; Mercé Jourdain; Philippe Jouvet; Anna Jung; Dafsah Juzar; Ouifiya Kafif; Florentia Kaguelidou; Neerusha Kaisbain; Thavamany Kaleesvran; Sabina Kali; Smaragdi Kalomoiri; Muhammad Aisar Ayadi Kamaluddin; Zul Amali Che Kamaruddin; Nadiah Kamarudin; Darshana Hewa Kandamby; Chris Kandel; Kong Yeow Kang; Pratap Karpayah; Christiana Kartsonaki; Daisuke Kasugai; Anant Kataria; Kevin Katz; Aasmine Kaur; Christy Kay; Lamees Kayyali; Seán Keating; Claire Kelly; Yvelynne Kelly; Andrea Kelly; Niamh Kelly; Aoife Kelly; Sadie Kelly; Maeve Kelsey; Ryan Kennedy; Kalynn Kennon; Maeve Kernan; Younes Kerroumi; Sharma Keshav; Imrana Khalid; Antoine Khalil; Coralie Khan; Irfan Khan; Michelle E Kho; Saye Khoo; Ryan Khoo; Denisa Khoo; Khor How Kiat; Yuri Kida; Peter Kiiza; Beathe Kiland Granerud; Anders Benjamin Kildal; Jae Burm Kim; Antoine Kimmoun; Nobuya Kitamura; Paul Klenerman; Rob Klont; Gry Kloumann Bekken; Stephen R Knight; Mamoru Komatsu; Volkan Korten; Arsène Kpangon; Karolina Krawczyk; Sudhir Krishnan; Vinothini Krishnan; Oksana Kruglova; Deepali Kumar; Ganesh Kumar; Bharath Kumar Tirupakuzhi Vijayaraghavan; Pavan Kumar Vecham; Dinesh Kuriakose; Ethan Kurtzman; Demetrios Kutsogiannis; Galyna Kutsyna; Konstantinos Kyriakoulis; Marie Lachatre; Marie Lacoste; John G. Laffey; Nadhem Lafhej; Marie Lagrange; Fabrice Laine; Olivier Lairez; Antonio Lalueza; Marc Lambert; Marie Langelot-Richard; Vincent Langlois; Eka Yudha Lantang; Marina Lanza; Cédric Laouénan; Samira Laribi; Delphine Lariviere; Stéphane Lasry; Odile Launay; Didier Laureillard; Yoan Lavie-Badie; Andy Law; Teresa Lawrence; Cassie Lawrence; Minh Le; Clément Le Bihan; Cyril Le Bris; Georges Le Falher; Lucie Le Fevre; Quentin Le Hingrat; Marion Le Maréchal; Soizic Le Mestre; Gwenaël Le Moal; Vincent Le Moing; Hervé Le Nagard; Paul Le Turnier; Todd C. Lee; Su Hwan Lee; James Lee; Jennifer Lee; Heng Gee Lee; Biing Horng Lee; Yi Lin Lee; Gary Leeming; Laurent Lefebvre; Bénédicte Lefebvre; Benjamin Lefèvre; Sylvie LeGac; Jean-Daniel Lelievre; François Lellouche; Adrien Lemaignen; Véronique Lemee; Anthony Lemeur; Gretchen Lemmink; Ha Sha Lene; Michela Leone; Marc Leone; Quentin Lepiller; François-Xavier Lescure; Olivier Lesens; Mathieu Lesouhaitier; Amy Lester-Grant; Sophie Letrou; Yves Levy; Bruno Levy; Claire Levy-Marchal; Erwan L'Her; Gianluigi Li Bassi; Janet Liang; Geoffrey Liegeon; Wei Shen Lim; Kah Chuan Lim; Chantre Lima; Bruno Lina; Lim Lina; Andreas Lind; Guillaume Lingas; Sylvie Lion-Daolio; Keibun Liu; Marine Livrozet; Patricia Lizotte; Navy Lolong; Leong Chee Loon; Diogo Lopes; Dalia Lopez-Colon; Anthony L. Loschner; Paul Loubet; Bouchra Loufti; Guillame Louis; Silvia Lourenco; Lee Low; Marije Lowik; Jia Shyi Loy; Jean Christophe Lucet; Carlos Lumbreras Bermejo; Carlos M. Luna; Olguta Lungu; Miles Lunn; Liem Luong; Nestor Luque; Dominique Luton; Ruth Lyons; Olavi Maasikas; Oryane Mabiala; Sara Machado; Moïse Machado; Gabriel Macheda; Hashmi Madiha; Guillermo Maestro de la Calle; Rafael Mahieu; Sophie Mahy; Lars S. Maier; Mylène Maillet; Thomas Maitre; Maximilian Malfertheiner; Nadia Malik; Paddy Mallon; Denis Malvy; Victoria Manda; Laurent Mandelbrot; Frank Manetta; Julie Mankikian; Edmund Manning; Aldric Manuel; Ceila Maria Sant`Ana Malaque; Flávio Marino; Samuel Markowicz; Ana Marques; Brian Marsh; Laura Marsh; Megan Marshal; John Marshall; Celina Turchi Martelli; Emily Martin; Guillaume Martin-Blondel; F. Eduardo Martinez; Ignacio Martin-Loeches; Martin Martinot; João Martins; Ana Martins; Caroline Martins Rego; Gennaro Martucci; Olga Martynenko; Eva Miranda Marwali; Marsilla Marzukie; David Maslove; Sabina Mason; Mohd Basri Mat Nor; Moshe Matan; Daniel Mathieu; Mathieu Mattei; Laurence Maulin; Michael Maxwell; Thierry Mazzoni; Lisa Mc Sweeney; Colin McArthur; Peter McCanny; Anne McCarthy; Aine McCarthy; Colin McCloskey; Rachael McConnochie; Sherry McDermott; Sarah E. McDonald; Aine McElroy; Samuel McElwee; Victoria McEneany; Natalie McEvoy; Allison McGeer; Chris McKay; Johnny McKeown; Kenneth A. McLean; Bairbre McNicholas; Elaine McPartlan; Edel Meaney; Cécile Mear-Passard; Maggie Mechlin; Omar Mehkri; Ferruccio Mele; Kusum Menon; France Mentré; Alexander J. Mentzer; Noémie Mercier; Emmanuelle Mercier; Antoine Merckx; Mayka Mergeay-Fabre; Blake Mergler; Laura Merson; António Mesquita; Osama Metwally; Agnès Meybeck; Dan Meyer; Alison M. Meynert; Vanina Meysonnier; Amina Meziane; Mehdi Mezidi; Céline Michelanglei; Isabelle Michelet; Efstathia Mihelis; Vladislav Mihnovit; Jennene Miller; Nor Arisah Misnan; Tahira Jamal Mohamed; Nik Nur Eliza Mohamed; Asma Moin; Elena Molinos; Brenda Molloy; Sinead Monahan; Mary Mone; Agostinho Monteiro; Claudia Montes; Giorgia Montrucchio; Shona C. Moore; Sarah Moore; Lina Morales Cely; Lucia Moro; Catherine Motherway; Ana Motos; Hugo Mouquet; Clara Mouton Perrot; Julien Moyet; Caroline Mudara; Ng Yong Muh; Dzawani Muhamad; Jimmy Mullaert; Fredrik Müller; Karl Erik Müller; Daniel Munblit; Aisling Murphy; Lorna Murphy; Patrick Murray; Marlène Murris; Srinivas Murthy; Himed Musaab; Gugapriyaa Muyandy; Dimitra Melia Myrodia; Dave Nagpal; Alex Nagrebetsky; Mangala Narasimhan; Nageswaran Narayanan; Nadège Neant; Coca Necsoi; Nikita Nekliudov; Raul Neto; Emily Neumann; Pauline Yeung Ng; Wing Yiu Ng; Anthony Nghi; Duc Nguyen; Orna Ni Choileain; Niamh Ni Leathlobhair; Alistair D Nichol; Prompak Nitayavardhana; Stephanie Nonas; Nurul Amani Mohd Noordin; Marion Noret; Nurul Faten Izzati Norharizam; Lisa Norman; Alessandra Notari; Mahdad Noursadeghi; Saad Nseir; Dwi Utomo Nusantara; Elsa Nyamankolly; Fionnuala O Brien; Annmarie O Callaghan; Annmarie O'Callaghan; Giovanna Occhipinti; Derbrenn OConnor; Takayuki Ogura; Sophie O'Halloran; Katie O'Hearn; Shinichiro Ohshimo; João Oliveira; Piero L. Olliaro; Conar O'Neil; David S.Y. Ong; Jee Yan Ong; Wilna Oosthuyzen; Anne Opavsky; Peter Openshaw; Claudia Milena Orozco-Chamorro; Jamel Ortoleva; Javier Osatnik; Linda O'Shea; Miriam O'Sullivan; Siti Zubaidah Othman; Nadia Ouamara; Rachida Ouissa; Eric Oziol; Maïder Pagadoy; Justine Pages; Massimo Palmarini; Giovanna Panarello; Prasan Kumar Panda; Lai Hui Pang; Mauro Panigada; Nathalie Pansu; Aurélie Papadopoulos; Rachael Parke; Jérémie Pasquier; Bruno Pastene; Fabian Patauner; Mohan Dass Pathmanathan; Patricia Patricio; Juliette Patrier; Lisa Patterson; Mical Paul; Christelle Paul; Jorge Paulos; William A. Paxton; Jean-François Payen; Sandra L Peake; Kalaiarasu Peariasamy; Miguel Pedrera Jiménez; Giles J. Peek; Florent Peelman; Nathan Peiffer-Smadja; Vincent Peigne; Mare Pejkovska; Paolo Pelosi; Ithan D. Peltan; Rui Pereira; Daniel Perez; Luis Periel; Thomas Perpoint; Antonio Pesenti; Vincent Pestre; Lenka Petrou; Michele Petrovic; Ventzislava Petrov-Sanchez; Frank Olav Pettersen; Gilles Peytavin; Scott Pharand; Michael Piagnerelli; Walter Picard; Olivier Picone; Carola Pierobon; Djura Piersma; Carlos Pimentel; Valentine Piquard; Catarina Pires; Isabelle Pironneau; Lionel Piroth; Ayodhia Pitaloka; Chiara Piubelli; Riinu Pius; Laurent Plantier; Hon Shen Png; Julien Poissy; Ryadh Pokeerbux; Sergio Poli; Georgios Pollakis; Diane Ponscarme; Diego Bastos Porto; Andra-Maris Post; Douwe F. Postma; Pedro Povoa; Diana Póvoas; Jeff Powis; Sofia Prapa; Sébastien Preau; Christian Prebensen; Jean-Charles Preiser; Anton Prinssen; Lucia Proença; Sravya Pudota; Oriane Puéchal; Bambang Pujo Semedi; Gregory Purcell; Luisa Quesada; Víctor Quirós González; Else Quist-Paulsen; Mohammed Quraishi; Fadi Qutishat; Christian Rabaud; Aldo Rafael; Marie Rafiq; Mutia Rahardjani; Amir Kamel Rahimi; Rozanah Abd Rahman; Ahmad Kashfi Haji Ab Rahman; Fernando Rainieri; Giri Shan Rajahram; Nagarajan Ramakrishnan; José Ramalho; Ahmad Afiq Ramli; Blandine Rammaert; Grazielle Viana Ramos; Ritika Ranjan; Christophe Rapp; Menaldi Rasmin; Indrek Rätsep; Tharmini Ravi; Andre Real; Stanislas Rebaudet; Sarah Redl; Brenda Reeve; Liadain Reid; Dag Henrik Reikvam; Renato Reis; Martine Remy; Hongru Ren; Anne-Sophie Resseguier; Matthieu Revest; Oleksa Rewa; Tiago Reyes; Luis Felipe Reyes; Maria Ines Ribeiro; David Richardson; Denise Richardson; Laurent Richier; Siti Nurul Atikah Ahmad Ridzuan; Ana L Rios; Asgar Rishu; Patrick Rispal; Karine Risso; Nicholas Rizer; Chiara Robba; André Roberto; Stephanie Roberts; David L. Robertson; Olivier Robineau; Ferran Roche-Campo; Paola Rodari; Simão Rodeia; Bernhard Roessler; Pierre-Marie Roger; Amanda Rojek; Juliette Romaru; Roberto Roncon-Albuquerque Jr.; Mélanie Roriz; Manuel Rosa-Calatrava; Michael Rose; Dorothea Rosenberger; Andrea Rossanese; Matteo Rossetti; Bénédicte Rossignol; Patrick Rossignol; Stella Rousset; Carine Roy; Benoît Roze; Clark D. Russell; Maria Ryan; Maeve Ryan; Steffi Ryckaert; Aleksander Rygh Holten; Isabela Saba; Musharaf Sadat; Valla Sahraei; Maximilien Saint-Gilles; Pranya Sakiyalak; Leonardo Salazar; Gabriele Sales; Stéphane Sallaberry; Charlotte Salmon Gandonniere; Hélène Salvator; Emely Sanchez; Olivier Sanchez; Angel Sanchez-Miralles; Vanessa Sancho-Shimizu; Gyan Sandhu; Zulfiqar Sandhu; Pierre-François Sandrine; Oana Sandulescu; Marlene Santos; Shirley Sarfo-Mensah; Iam Claire E. Sarmiento; Benjamine Sarton; Sree Satyapriya; Rumaisah Satyawati; Egle Saviciute; Yen Tsen Saw; Justin Schaffer; Tjard Schermer; Arnaud Scherpereel; Marion Schneider; Stephan Schroll; Michael Schwameis; Janet T. Scott; James Scott-Brown; Nicholas Sedillot; Tamara Seitz; Mageswari Selvarajoo; Caroline Semaille; Malcolm G. Semple; Rasidah Bt Senian; Eric Senneville; Claudia Sepulveda; Filipa Sequeira; Tânia Sequeira; Ary Serpa Neto; Pablo Serrano Balazote; Ellen Shadowitz; Syamin Asyraf Shahidan; Mohammad Shamsah; Anuraj Shankar; Shaikh Sharjeel; Pratima Sharma; Catherine A. Shaw; Victoria Shaw; John Robert Sheenan; Haixia Shi; Nobuaki Shime; Hiroaki Shimizu; Keiki Shimizu; Sally Shrapnel; Hoi Ping Shum; Nassima Si Mohammed; Ng Yong Siang; Jeanne Sibiude; Atif Siddiqui; Louise Sigfrid; Piret Sillaots; Catarina Silva; Rogério Silva; Maria Joao Silva; Benedict Sim Lim Heng; Wai Ching Sin; Punam Singh; Budha Charan Singh; Pompini Agustina Sitompul; Karisha Sivam; Vegard Skogen; Sue Smith; Benjamin Smood; Coilin Smyth; Michelle Smyth; Morgane Snacken; Dominic So; Tze Vee Soh; Joshua Solomon; Tom Solomon; Emily Somers; Agnès Sommet; Rima Song; Myung Jin Song; Tae Song; Jack Song Chia; Albert Sotto; Edouard Soum; Marta Sousa; Ana Chora Sousa; Maria Sousa Uva; Vicente Souza-Dantas; Alexandra Sperry; Elisabetta Spinuzza; Shiranee Sriskandan; Sarah Stabler; Thomas Staudinger; Stephanie-Susanne Stecher; Trude Steinsvik; Ymkje Stienstra; Birgitte Stiksrud; Eva Stolz; Amy Stone; Adrian Streinu-Cercel; Anca Streinu-Cercel; Ami Stuart; David Stuart; Richa Su; Decy Subekti; Gabriel Suen; Jacky Y. Suen; Prasanth Sukumar; Asfia Sultana; Charlotte Summers; Dubravka Supic; Deepashankari Suppiah; Magdalena Surovcová; Suwarti; Andrey Svistunov; Sarah Syahrin; Konstantinos Syrigos; Jaques Sztajnbok; Konstanty Szuldrzynski; Shirin Tabrizi; Fabio S. Taccone; Lysa Tagherset; Shahdattul Mawarni Taib; Sara Taleb; Jelmer Talsma; Maria Lawrensia Tampubolon; Kim Keat Tan; Yan Chyi Tan; Hiroyuki Tanaka; Taku Tanaka; Hayato Taniguchi; Coralie Tardivon; Pierre Tattevin; M Azhari Taufik; Hassan Tawfik; Richard S. Tedder; Tze Yuan Tee; João Teixeira; Marie-Capucine Tellier; Sze Kye Teoh; Vanessa Teotonio; François Téoulé; Pleun Terpstra; Olivier Terrier; Nicolas Terzi; Hubert Tessier-Grenier; Adrian Tey; Alif Adlan Mohd Thabit; Zhang Duan Tham; Suvintheran Thangavelu; Elmi Theron; Vincent Thibault; Simon-Djamel Thiberville; Benoît Thill; Jananee Thirumanickam; Shaun Thompson; David Thomson; Emma C. Thomson; Mathew Thorpe; Surain Raaj Thanga Thurai; Ryan S. Thwaites; Paul Tierney; Vadim Tieroshyn; Peter S Timashev; Jean-François Timsit; Noémie Tissot; Fiona Toal; Jordan Zhien Yang Toh; Maria Toki; Kristian Tonby; Sia Loong Tonnii; Marta Torre; Antoni Torres; Hernando Torres-Zevallos; Michael Towers; Tony Trapani; Cécile Tromeur; Ioannis Trontzas; Tiffany Trouillon; Jeanne Truong; Christelle Tual; Sarah Tubiana; Helen Tuite; Alexis F. Turgeon; Jean-Marie Turmel; Lance C.W. Turtle; Anders Tveita; Pawel Twardowski; Andrew Udy; Roman Ullrich; Alberto Uribe; Asad Usman; Timothy M. Uyeki; Cristinava Vajdovics; Luís Val-Flores; Amélie Valran; Stijn Van de Velde; Marcel van den Berge; Job van der Palen; Paul van der Valk; Nicky Van Der Vekens; Peter Van der Voort; Sylvie Van Der Werf; Laura van Gulik; Jarne Van Hattem; Carolien van Netten; Ilonka van Veen; Noémie Vanel; Henk Vanoverschelde; Pooja Varghese; Michael Varrone; Shoban Raj Vasudayan; Charline Vauchy; Shaminee Veeran; Aurélie Veislinger; Sebastian Vencken; Sara Ventura; Annelies Verbon; José Ernesto Vidal; César Vieira; Joy Ann Villanueva; Andrea Villoldo; Benoit Visseaux; Chiara Vitiello; Harald Vonkeman; Fanny Vuotto; Noor Hidayu Wahab; Suhaila Abdul Wahab; Nadirah Abdul Wahid; Marina Wainstein; Laura Walsh; Chih-Hsien Wang; Steve Webb; Jia Wei; Tan Pei Wen; Sanne Wesselius; Murray Wham; Bryan Whelan; Nicole White; Paul Henri Wicky; Aurélie Wiedemann; Surya Otto Wijaya; Keith Wille; Sue Willems; Bailey Williams; Virginie Williams; Patricia J Williams; Evert-Jan Wils; Jessica Wittman; Calvin Wong; Xin Ci Wong; Yew Sing Wong; Teck Fung Wong; Gan Ee Xian; Lim Saio Xian; Pei Xuan Kuan; Ioannis Xynogalas; Siti Rohani Binti Mohd Yakop; Masaki Yamazaki; Elizabeth Yarad; Yazdan Yazdanpanah; Nicholas Yee Liang Hing; Cécile Yelnik; Chian Hui Yeoh; Stephanie Yerkovich; Toshiki Yokoyama; Hodane Yonis; Obada Yousif; Akram Zaaqoq; Marion Zabbe; Masliza Zahid; Maram Zahran; Nor Zaila Binti Zaidan; Maria Zambon; Miguel Zambrano; Alberto Zanella; Nurul Zaynah; Hiba Zayyad; Alexander Zoufaly; David Zucman.

## Supplementary Materials

Supplementary File (PDF)

**Supplementary Statement S1.** Study ethics approval.

**Supplementary Table S1.** STROBE Statement - checklist of items that should be included in reports of observational studies.

**Supplementary Table S2.** Definitions used for clinical COVID-19.

**Supplementary Table S3.** Definition of comorbidities, complications, and outcomes from the ISARIC case report forms (CRF).

**Supplementary Table S4.** Missingness (%) from variables in Table 1.

**Supplementary Figure S1.** Breakdown by country income level and individual country of patients excluded on the basis of (A) not having an ICU admission or (B) having less than 2 serum creatinine measurements.

**Supplementary Figure S2.** Breakdown of patients with AKI by individual country.

**Supplementary Figure S3.** Day of peak AKI by country income level.

**Supplementary Figure S4.** Community vs. hospital-acquired AKI based on 48 hour cut-off from admission by individual country.

**Supplementary Figure S5.** Admission treatments and outcomes stratified by AKI status and country income level.
