# Supplementary material for: Association of Country Income Level With the Characteristics and Outcomes of Critically Ill Patients Hospitalized With Acute Kidney Injury and COVID-19
Source: Kidney Int Rep. 2023 May 27;8(8):1514–30. doi: 10.1016/j.ekir.2023.05.015 (PMC10219675; doi:10.1016/j.ekir.2023.05.015)

TaSupporting Material

Statement S1. Study ethics approval

Ethics Committee approval for this work was given by the World Health Organisation Ethics Review Committee (RPC571 and RPC572 on 25 April 2013). Institutional approval was additionally obtained by participating sites including the South Central Oxford C Research Ethics Committee in England (Ref 13/SC/0149) and the Scotland A Research Ethics Committee (Ref 20/SS/0028) for the United Kingdom and the Human Research Ethics Committee (Medical) at the University of the Witwatersrand in South Africa as part of a national surveillance programme (M160667) collectively representing the majority of the data. Other institutional and national approvals are in place as per local requirements.

Table S1 STROBE Statement - checklist of items that should be included in reports of observational studies

	Item No.	Recommendation	Page No.	Relevant text from manuscript
**Title and abstract**	1	(*a*) Indicate the study's design with a commonly used term in the title or the abstract	3	
		(*b*) Provide in the abstract an informative and balanced summary of what was done and what was found	3	
**Introduction**				
Background/rationale	2	Explain the scientific background and rationale for the investigation being reported	4	
Objectives	3	State specific objectives, including any prespecified hypotheses	4	"…we hypothesized that…"
**Methods**				
Study design	4	Present key elements of study design early in the paper	5	ISARIC Clinical Characterization protocol (p 5)
Setting	5	Describe the setting, locations, and relevant dates, including periods of recruitment, exposure, follow-up, and data collection	5-6	"…admitted to hospital from January 30th…"
Participants	6	(*a*) *Cohort study*—Give the eligibility criteria, and the sources and methods of selection of participants. Describe methods of follow-up<br>*Case-control study*—Give the eligibility criteria, and the sources and methods of case ascertainment and control selection. Give the rationale for the choice of cases and controls<br>*Cross-sectional study*—Give the eligibility criteria, and the sources and methods of selection of participants	5-6	Inclusion & Exclusion heading
		(*b*) *Cohort study*—For matched studies, give matching criteria and number of exposed and unexposed<br>*Case-control study*—For matched studies, give matching criteria and the number of controls per case		
Variables	7	Clearly define all outcomes, exposures, predictors, potential confounders, and effect modifiers. Give diagnostic criteria, if applicable	5-7, S1 Table & 2, table 1	
Data sources/ measurement	8*	For each variable of interest, give sources of data and details of methods of assessment (measurement). Describe comparability of assessment methods if there is more than one group	S2 Table	
Bias	9	Describe any efforts to address potential sources of bias	5-6	"…we excluded patients who had not been admitted to the ICU…"
Study size	10	Explain how the study size was arrived at	p 7 and Fig 1	

Quantitative variables	11	Explain how quantitative variables were handled in the analyses. If applicable, describe which groupings were chosen and why	5-6 Fig 1
Statistical methods	12	(*a*) Describe all statistical methods, including those used to control for confounding	6-7
		(*b*) Describe any methods used to examine subgroups and interactions	6-7
		(*c*) Explain how missing data were addressed	6-7 "all variables were analysed but…"
		(*d*) *Cohort study*—If applicable, explain how loss to follow-up was addressed<br>*Case-control study*—If applicable, explain how matching of cases and controls was addressed<br>*Cross-sectional study*—If applicable, describe analytical methods taking account of sampling strategy	5-6
		(*e*) Describe any sensitivity analyses	
**Results**			
Participants	13*	(a) Report numbers of individuals at each stage of study—eg numbers potentially eligible, examined for eligibility, confirmed eligible, included in the study, completing follow-up, and analysed	8
		(b) Give reasons for non-participation at each stage	
		(c) Consider use of a flow diagram	Fig 1
Descriptive data	14*	(a) Give characteristics of study participants (eg demographic, clinical, social) and information on exposures and potential confounders	8-9 & table 1
		(b) Indicate number of participants with missing data for each variable of interest	S3 Table
		(c) *Cohort study*—Summarise follow-up time (eg, average and total amount)	5 "data were collected and analyzed for the duration…"
Outcome data	15*	*Cohort study*—Report numbers of outcome events or summary measures over time	Table 1
		Case-control study—Report numbers in each exposure category, or summary measures of exposure	
		Cross-sectional study—Report numbers of outcome events or summary measures	
Main results	16	(*a*) Give unadjusted estimates and, if applicable, confounder-adjusted estimates and their precision (eg, 95% confidence interval). Make clear which confounders were adjusted for and why they were included	Table 2, page 7 & 8-9
		(*b*) Report category boundaries when continuous variables were categorized	Table 2
		(*c*) If relevant, consider translating estimates of relative risk into absolute risk for a meaningful time period	

Continued on next page

Other analyses	17	Report other analyses done—eg analyses of subgroups and interactions, and sensitivity analyses	
**Discussion**			
Key results	18	Summarise key results with reference to study objectives	9
Limitations	19	Discuss limitations of the study, taking into account sources of potential bias or imprecision. Discuss both direction and magnitude of any potential bias	10
Interpretation	20	Give a cautious overall interpretation of results considering objectives, limitations, multiplicity of analyses, results from similar studies, and other relevant evidence	9-11
Generalisability	21	Discuss the generalisability (external validity) of the study results	11
**Other information**			
Funding	22	Give the source of funding and the role of the funders for the present study and, if applicable, for the original study on which the present article is based	11

*Give information separately for cases and controls in case-control studies and, if applicable, for exposed and unexposed groups in cohort and cross-sectional studies.

Table S2. Definitions used for clinical COVID-19

Source of definition	Definition for clinical COVID-19
World Health Organization (WHO)	A combination of acute fever and cough,<br><br>Or<br><br>A combination of three or more of: fever, cough, general weakness and fatigue, headache, myalgia, sore throat, coryza, dyspnoea, anorexia, nausea and vomiting, diarrhoea, altered mental status
Centers for Disease Control (CDC), United States	At least two of: fever, chills*, rigors *, myalgia, headache, sore throat, new olfactory and taste disorder,<br><br>Or<br><br>At least one of: cough, shortness of breath, difficulty breathing*
Public Health England	New cough, or temperature 37.8°C, or a loss or change in sense of smell or taste
European Center for Disease Prevention and Control	At least one of: cough, fever, shortness of breath, sudden onset anosmia, ageusia or dysgeusia

* Symptom information not collected in the Case Report Form (CRF)

Table S3. Definition of comorbidities, complications and outcomes from the ISARIC case report forms (CRF)

Comorbidity	Definition
Chronic cardiac disease	Any of coronary artery disease, heart failure, congenital heart disease, cardiomyopathy, rheumatic heart disease.
Hypertension	Elevated arterial blood pressure diagnosed clinically, >140mmHg systolic or >90mmHg diastolic.
Chronic pulmonary disease (not asthma)	Any of chronic obstructive pulmonary disease (chronic bronchitis, chronic obstructive pulmonary disease (COPD), emphysema), cystic fibrosis, bronchiectasis, interstitial lung disease, pre-existing requirement for long term oxygen therapy.
Chronic kidney disease	Clinician-diagnosed chronic kidney disease, chronic estimated glomerular filtration rate < 60 $mL/min/1.73m^2$, history of kidney transplantation
Obesity	Patients for whom an attending clinician has assessed them to be obese - ideally but not necessarily with an objective measurement of obesity, such as calculation of the body mass index (BMI of 30 kg/m^2 or more) or measurement of abdominal girth.
Liver disease (mild, moderate & severe)	Cirrhosis with or without portal hypertension or chronic hepatitis, with or without bleeding or a history of variceal bleeding
Type 2 Diabetes	Clinician diagnosed requiring oral or subcutaneous treatment
Dementia	Clinical diagnosis of dementia
Malnutrition	Any clinically identified deficiency in intake, either of total energy or of specific nutrients that led to a dietetic intervention or referral prior to the onset of COVID-19 symptoms. Does not include people who needed supplementary nutrition solely due to reduced intake during their current illness episode.
Complication	
Bacterial pneumonia	Clinically or radiologically diagnosed bacterial pneumonia (including community, hospital and ventilator acquired) managed with antimicrobials. Bacteriological confirmation not required.
Cardiac arrest	Sudden cessation of cardiac activity with no normal breathing and no signs of circulation.
Coagulation disorder	Abnormal coagulation identified by abnormal prothrombin time or activated partial thromboplastin time. Disseminated intravascular coagulation (DIC; consumption coagulopathy; defibrination syndrome) is defined by thrombocytopenia, prolonged prothrombin time, low fibrinogen, elevated D-dimer and thrombotic microangiopathy.
Rhabdomyolysis	Rhabdomyolysis is a syndrome characterised by muscle necrosis and the release of myoglobin into the blood. Muscle biopsy, electromyography, radiological imaging and the presence of myoglobinuria are not required for the diagnosis.

Acute kidney injury	Acute kidney injury is defined as any of:<br>• Increase in serum creatinine by ≥0.3 mg/dL (≥26.5 µmol/L) within 48 hours<br>• Increase in serum creatinine to ≥1.5 times baseline, which is known or presumed to have occurred within the prior 7 days<br>• Urine volume <0.5 mL/kg/hour for 6 hours
Outcomes	
ICU admission	Admission to an intensive care unit (ICU) or high dependency care unit (HDU)
Invasive mechanical ventilation	Any mechanical ventilation delivered following intubation or via a tracheostomy. Does not include patients who are breathing independently via a tracheostomy.
Length of stay	Number of days in hospital up until discharge or study censoring date
Still in hospital	Patient is still in hospital at the time of the study censoring date
Transferred	Patient has been transferred to another facility that provides medical care. This could be a specialist center for more intensive treatment or a step-down for rehabilitation. It does not include facilities that solely provide social care (these patients should be listed as discharged alive).
Discharged alive	Patient has been discharged to their usual place of residence before their illness, to the home of a relative or friend, or to a social care facility, because their illness is no longer severe enough to warrant treatment in a medical facility.
Death	Patient died in the hospital

Figure S1 Breakdown by country income level and individual country of patients excluded on the basis of (A) not having an ICU admission or (B) having less than 2 serum creatinine measurements.

A.

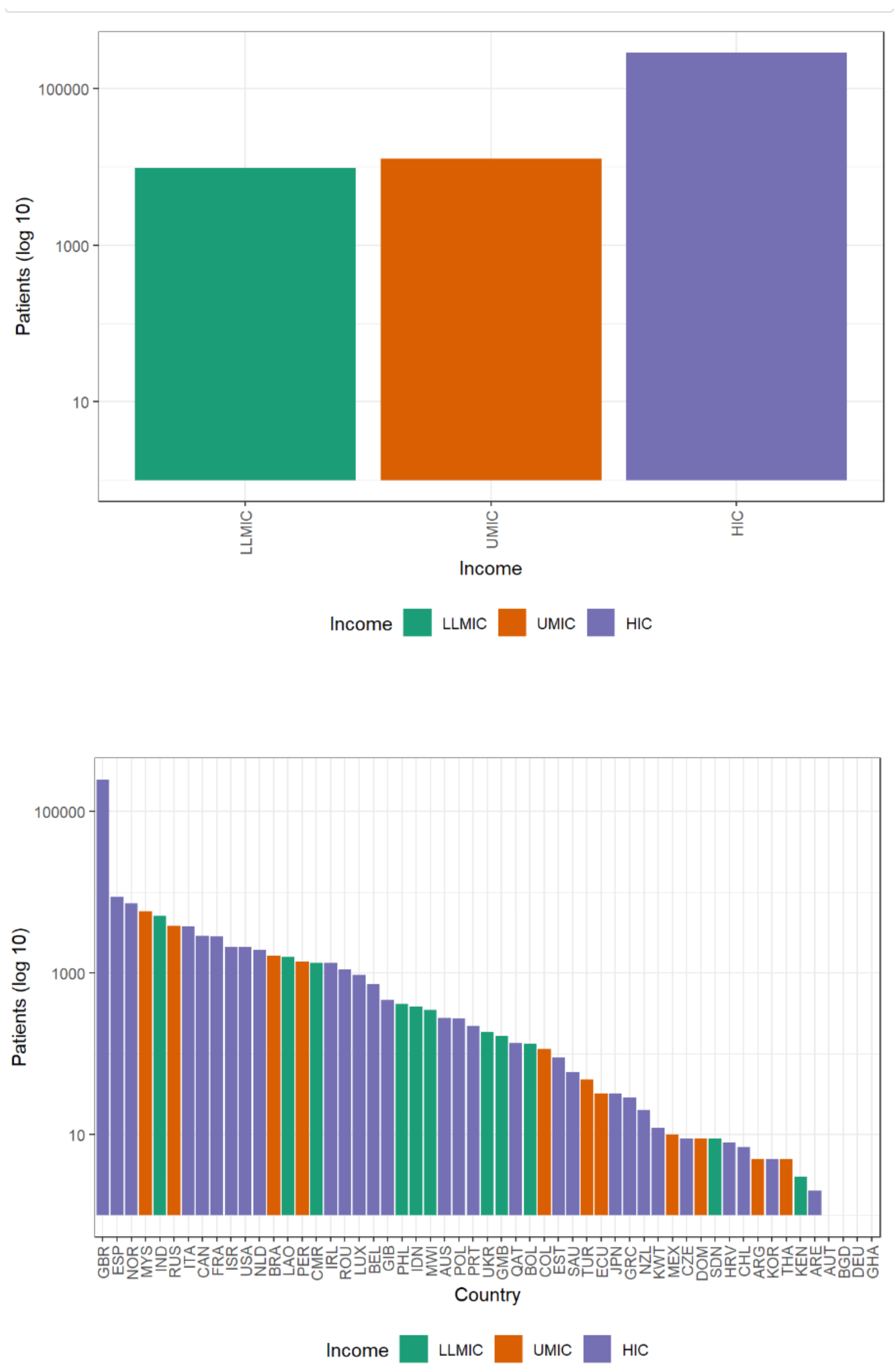

B.

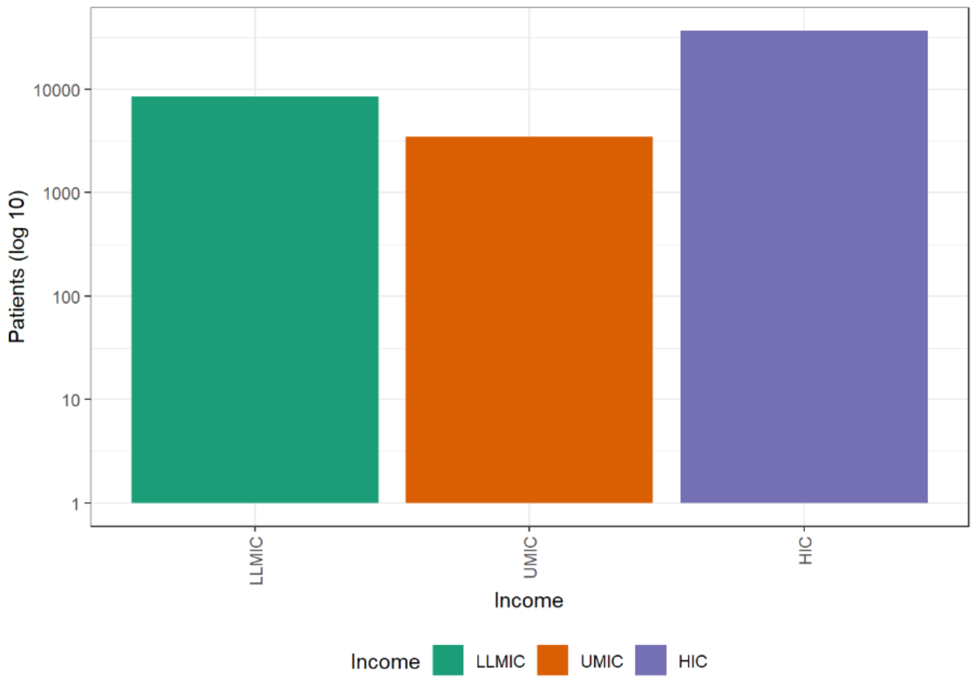
Patients (log 10)
10000
1000
100
10
1
LLMIC
UMIC
HIC
Income
Income
LLMIC
UMIC
HIC

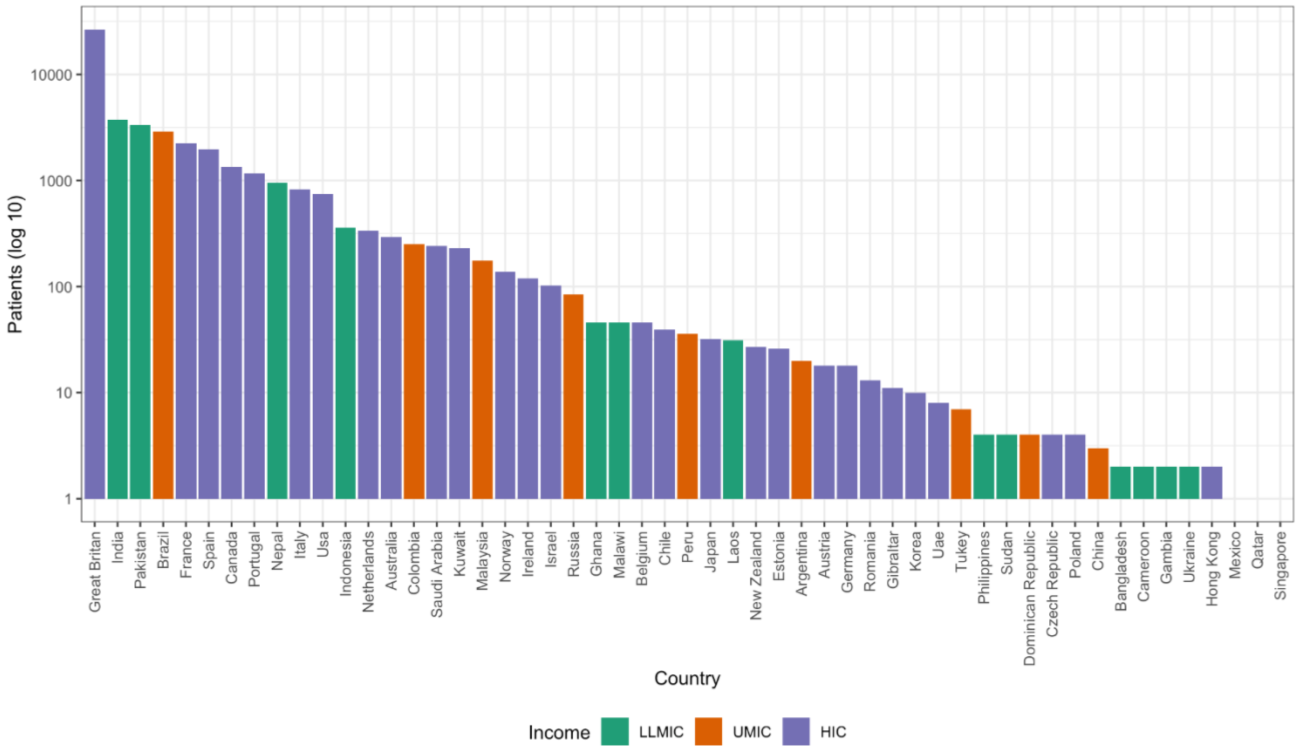
Patients (log 10)
10000
1000
100
10
1
Great Britan
India
Pakistan
Brazil
France
Spain
Canada
Portugal
Nepal
Italy
Usa
Indonesia
Netherlands
Australia
Colombia
Saudi Arabia
Kuwait
Malaysia
Norway
Ireland
Israel
Russia
Ghana
Malawi
Belgium
Chile
Peru
Japan
Laos
New Zealand
Estonia
Argentina
Austria
Germany
Romania
Gibraltar
Korea
Uae
Tukey
Philippines
Sudan
Dominican Republic
Czech Republic
Poland
China
Bangladesh
Cameroon
Gambia
Ukraine
Hong Kong
Mexico
Qatar
Singapore
Country
Income
LLMIC
UMIC
HIC

Table S4 Missingness (%) from variables in Table 1

		LLMIC	UMIC	HIC
**Demographics**				
	Age, yr, median (IQR)	0	0	0
	Female (%)	0	1	0
**AKI Grades & RRT, n (%)**				
	AKI grade 1	0	0	0
	AKI grade 2	0	0	0
	AKI grade 3 with RRT	0	8	0
	AKI grade 3 w/o RRT	0	0	0
	RRT	0	8	0
**Comorbidities**, n (%)**				
	Chronic Kidney Disease	1	4	5
	Chronic Cardiac Disease	1	3	4
	Chronic Pulmonary Disease	1	4	5
	Hypertension	1	3	21
	Dementia	1	8	13
	Liver Disease	1	5	5
	Malnutrition	1	5	11
	Obesity	1	9	18
**Signs & Symptoms on Admission, n (%)**				
	Altered consciousness/confusion	1	18	15
	Diarrhoea	1	11	15
	Fever	1	11	8
	Vomiting/nausea	1	11	15
	Muscle aches/joint pain	1	12	18
	Headache	1	12	19
	Cough	1	11	7
	Shortness of breath	1	9	5
**Observations on Admission, median (IQR)**				
	Temperature, C	3	12	5
	Systolic BP, mmHg	1	11	5
	Diastolic BP, mmHg	1	12	6
	Heart rate, BPM	3	18	5

	Respiratory rate, per min	3	14	9
	Oxygen saturation, %	3	12	6
**Laboratory Results on Admission, median (IQR)**				
	Potassium (mmol/L)	3	12	11
	sCr (µmol/L)	2	7	9
	eGFR (ml/min/1.73m2)	0	1	0
	Hemoglobin (g/L)	2	8	10
	Sodium (mmol/L)	4	13	9
**Admission Treatment, n (%)**				
	Antiviral and COVID-19 targeting agents	1	17	5
	Antibiotic agents	1	16	3
	Antifungal agents	1	10	9
	Corticosteroids	1	9	5
	Invasive mechanical ventilation	0		1
**Complications**, n (%)**				
	Bacterial pneumonia	1	13	17
	Cardiac arrest	1	16	8
	Coagulation disorder	1	18	17
	Rhabdomyolysis	1	19	17
**Outcomes, n (%)**				
	Transferred	0	0	0
	Discharged	0	0	0
	Death	0	0	0
	Length of Stay (median, IQR)	0	3	18

LLMIC: low- and lower-middle income countries; UMIC: Upper-middle income countries; HIC: high income countries

Figure S2 - Breakdown of patients with AKI by individual country*

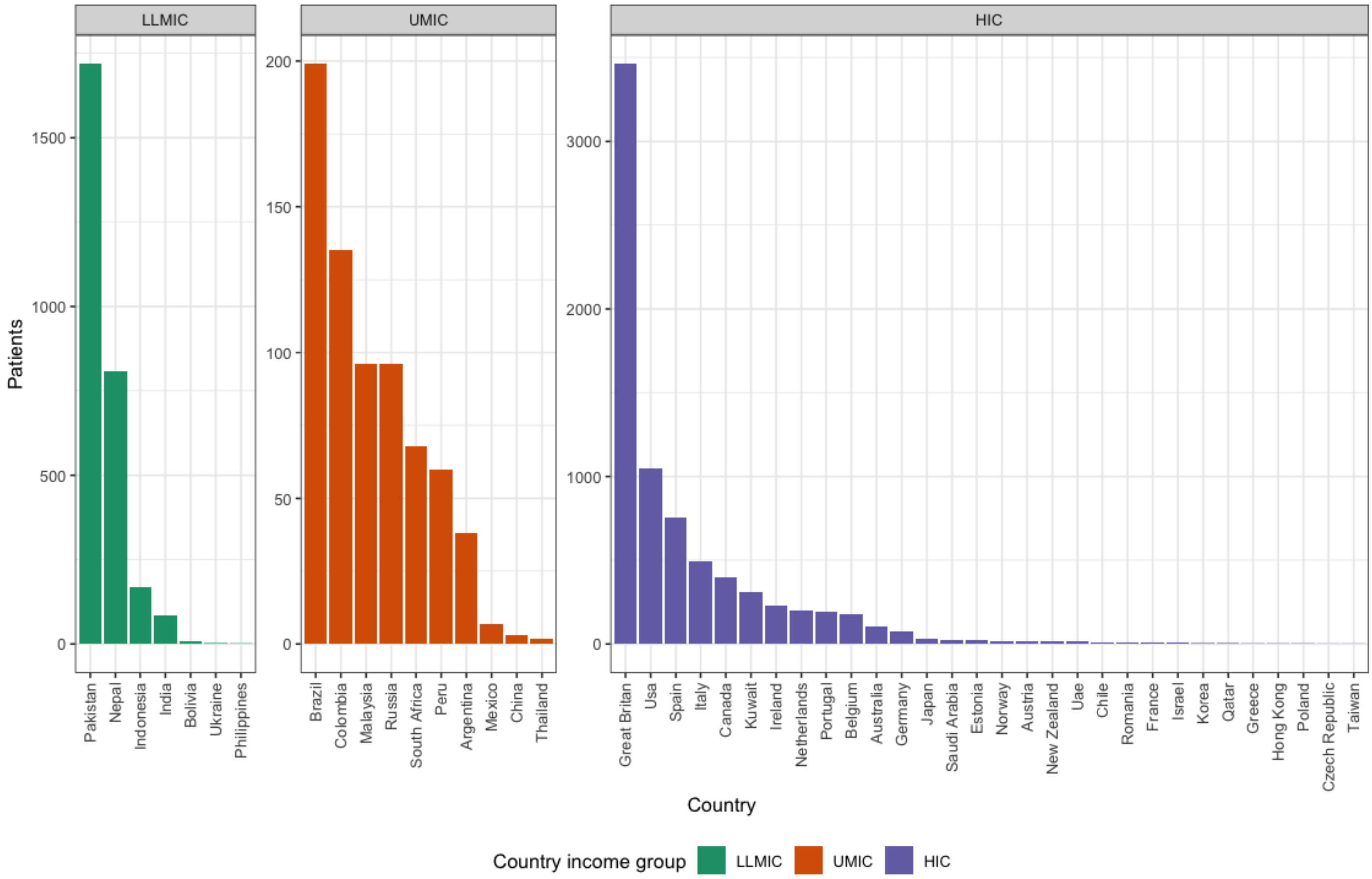

* patient scales differ between income groups.

Figure S3 – Day of peak AKI by country income level

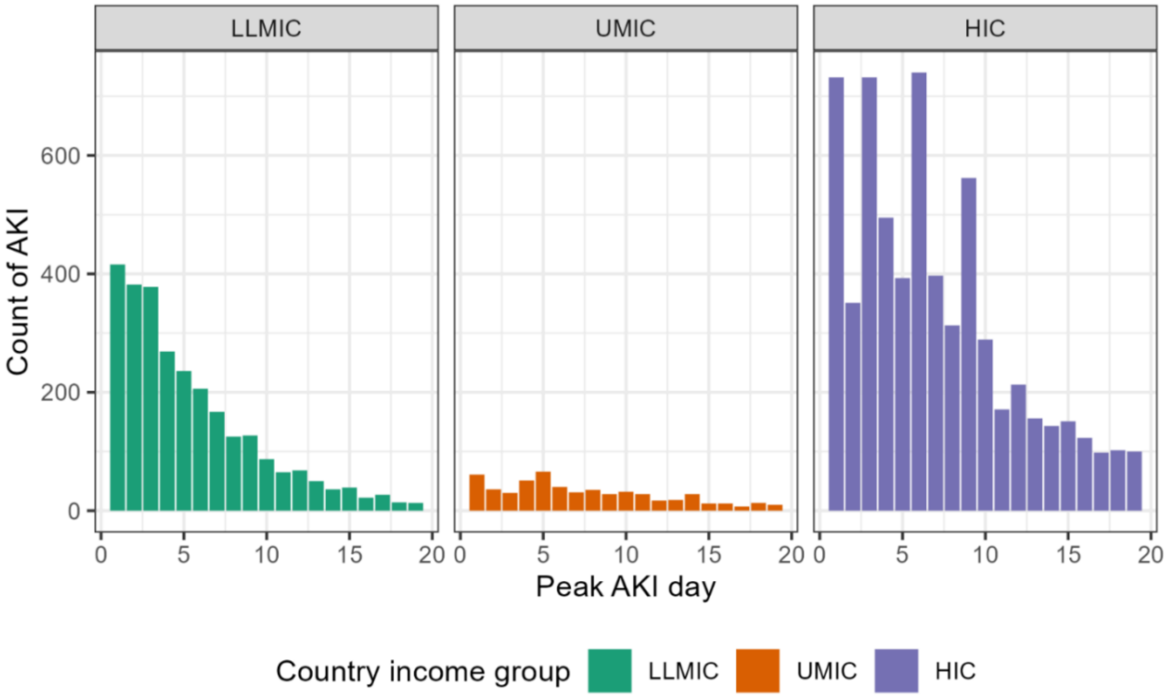

Figure S4 - Community- vs hospital-acquired AKI based on 48 hr cut-off from admission by individual country*

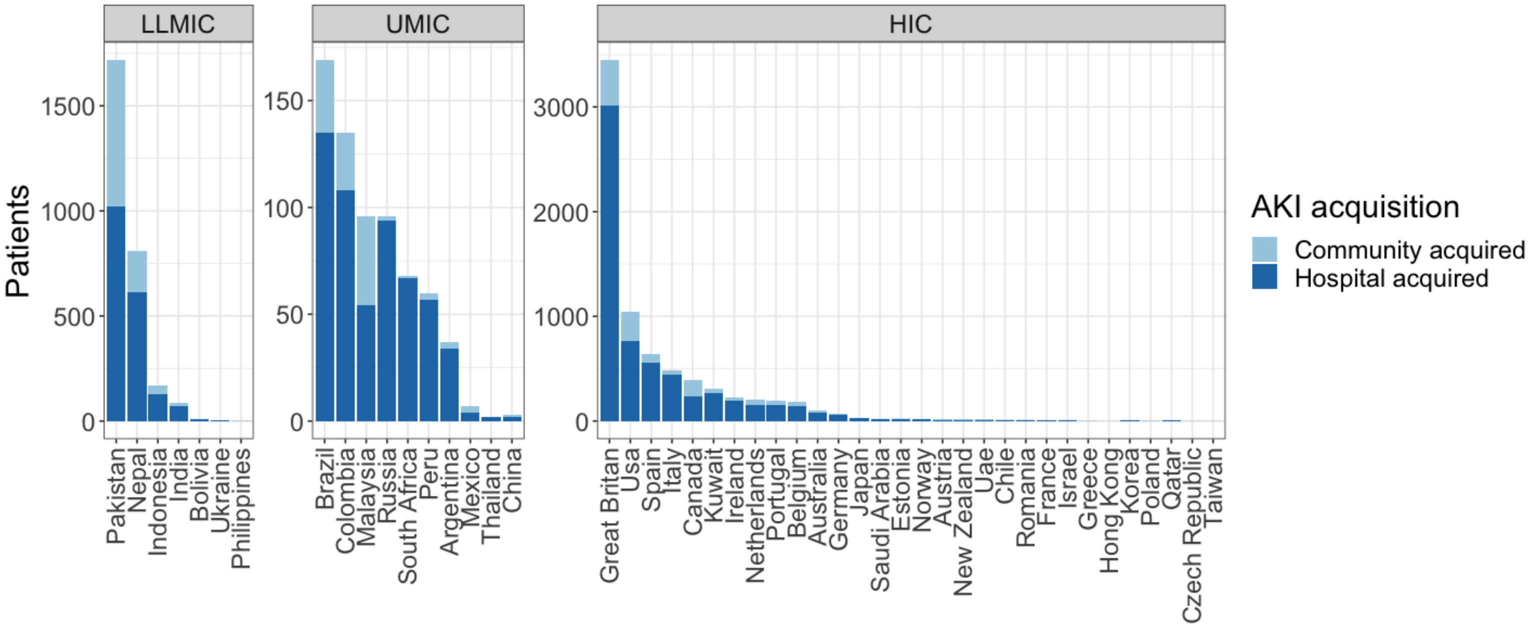

*note: patient scales differ between income groups.

Figure S5 - Admission treatments and outcomes stratified by AKI status and country income level

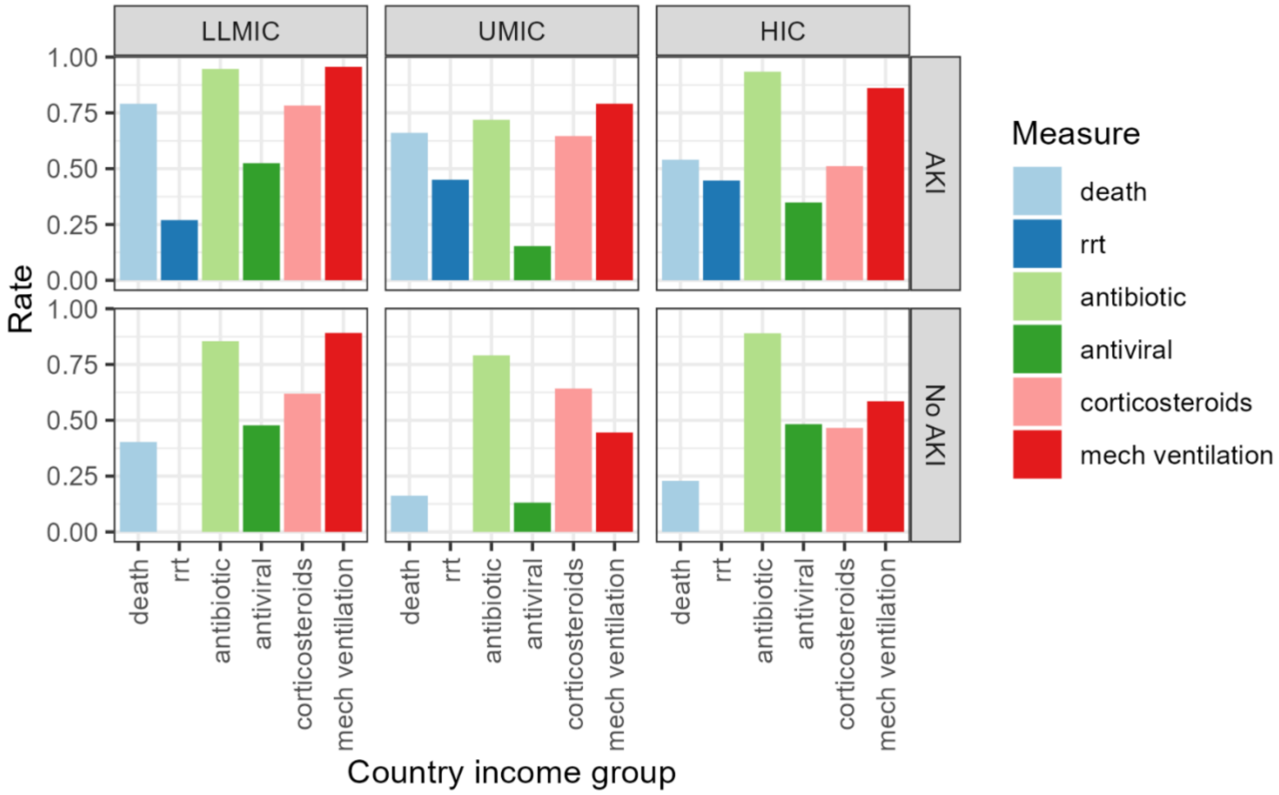

Supplement: Supplementary File (PDF) [file mmc1.pdf]
